# Supplementary material for: A cysteine-less and ultra-fast split intein rationally engineered from being aggregation-prone to highly efficient in protein trans-splicing
Source: Nat Commun. 2025 Mar 19;16:2723. doi: 10.1038/s41467-025-57596-x (PMC11923092; doi:10.1038/s41467-025-57596-x)
Supplement: Supplementary file 1 — Supplementary Information [file 41467_2025_57596_MOESM1_ESM.pdf]

Supplementary Information

for

A Cysteine-Less and Ultra-Fast Split Intein Rationally Engineered from Being Aggregation-Prone to Highly Efficient in Protein *trans*-Splicing

Christoph Humberg[1], Zahide Yilmaz[1], Katharina Fitzian[1], Wolfgang Dörner[1], Daniel Kümmel[1], Henning D. Mootz[1]*

[1]Institute of Biochemistry, University of Münster, Correnssstraße 36, 48149 Münster, Germany

eMail: Henning.Mootz@uni-muenster.de

Table of Contents Page

**Suppl. Note 1**:	Biophysical and kinetical investigation of the underlying assembly mechanism by biolayer interferometry.	S10
**Suppl. Note 2**:	Crystal structure of the Aes123 PolB1 intein.	S17
**Suppl. Table 1**:	List of purified recombinant protein constructs and their expression plasmids.	S2
**Suppl. Table 2**:	List of plasmids used for mammalian cell culture.	S3
**Suppl. Table 3**:	Kinetical binding parameters of the Aes123 PolB1 intein.	S9
**Suppl. Table 4**:	Crystallographic data collection and structure refinement.	S15
**Suppl. Table 5**:	Inteins and the corresponding canonical Int^N sequences used to Compare the predicted aggregation tendency.	S27
**Suppl. Figure 1**:	Intein sequence features and protein splicing mechanism.	S4
**Suppl. Figure 2**:	The C-terminal precursor is completely consumed when the N-terminal precursor is given in excess.	S5
**Suppl. Figure 3**:	Collective refolding of both split intein precursor proteins leads to quantitative splicing.	S5
**Suppl. Figure 4**:	Aes^N fragment aggregation is independent of the extein context, the expression system and ionic strength.	S6
**Suppl. Figure 5**:	Investigation of the Aes split intein assembly mechanism by biolayer interferometry (BLI).	S7
**Suppl. Figure 6**:	Investigation of the underlying relationship between the Aes assembly and splice mechanism.	S8
**Suppl. Figure 7**:	Sequence-based prediction of the folding state by charge-hydrophobicity plot and amino acid composition profiling.	S11
**Suppl. Figure 8**:	Splice activity of the Aes^N precursor with a short N-terminal extein of only 3 residues.	S11
**Suppl. Figure 9**:	Biophysical investigation of the Aes^N precursor.	S12
**Suppl. Figure 10**:	Principle of the photoreactive carbene labeling and LC-MS based footprinting.	S13
**Suppl. Figure 11**:	SEC analysis of the single Aes^N fragment segments fused to MBP	S14
**Suppl. Figure 12**:	Structural analysis of the catalytic center and the charge/hydrophobicity distribution among the intein fragments using the crystal structure of the Aes123 PolB1 intein	S16
**Suppl. Figure 13**:	Structural comparison between the cysteine-less Aes123 PolB1 and the PolB16 inteins.	S16
**Suppl. Figure 14**:	Portion of the electron density map of the Aes intein crystal structure	S18
**Suppl. Figure 15**:	Influence of the aggregation-reducing mutations on the extent of side reactionsand on the folding cooperativity to the canonical intein complex structure.	S19
**Suppl. Figure 16**:	Analysis of the aggregation-reducing mutants of MBP-Aes^N-H_6.	S20
**Suppl. Figure 17**:	The Aes123 PolB1 triple mutant increases the expression yield and protein purity.	S22
**Suppl. Figure 18**:	Intein-mediated generation of a bispecific nanobody-dimer for cell surface labelling.	S23
**Suppl. Figure 19**:	Nanobody functionalization using intein-mediated dual thiol-bioconjugation.	S24
**Suppl. Figure 20**:	*In silico* aggregation-prone site prediction of commonly used inteins.	S25
**Suppl. Figure 21**:	Int^N aggregation into inactive species in the natively split *Npu* DnaE intein.	S28
**Suppl. Figure 22**:	SEC analysis of the purified and isolated monomeric or aggregated proteins used throughout this study.	S28
**Suppl. Figure 23**:	Analytics to the used constructs.	S29

Supplementary Tables, Figures and Notes

Supplementary Table 1 List of purified recombinant protein constructs and their expression plasmids.

Protein	Construct	Encoding Plasmid	Vector System	Reference
**1P**	MBP-YIDTD-Aes^N-H_6	pTT04	pMAL-c2x	Ref[1]
**1P**(I7Q)	MBP-Aes^N(I7Q)-H_6	pCH96	pMAL-c2x	this work
**1P**(Y64A)	MBP-Aes^N(Y64A)-H_6	pCH141	pMAL-c2x	this work
**1P**(Y64N)	MBP-Aes^N(Y64N)-H_6	pCH127	pMAL-c2x	this work
**1P**(Y64K)	MBP-Aes^N(Y64K)-H_6	pCH126	pMAL-c2x	this work
**1P**(I65G)	MBP-Aes^N(I65G)-H_6	pCH87	pMAL-c2x	this work
**1P**(I65A)	MBP-Aes^N(I65A)-H_6	pCH95	pMAL-c2x	this work
**1P**(T69K)	MBP-Aes^N(T69K)-H_6	pCH18	pMAL-c2x	this work
**1P**(F75H)	MBP-Aes^N(F75H)-H_6	pCH128	pMAL-c2x	this work
**1P**(V84A)	MBP-Aes^N(V84A)-H_6	pCH121	pMAL-c2x	this work
**1P**(I85K)	MBP-Aes^N(I85K)-H_6	pCH125	pMAL-c2x	this work
**1P**(I101K)	MBP-Aes^N(I101K)-H_6	pCH124	pMAL-c2x	this work
**1P**(V115G)	MBP-Aes^N(V115G)-H_6	pCH90	pMAL-c2x	this work
**1P**(W117K)	MBP-Aes^N(W117K)-H_6	pCH91	pMAL-c2x	this work
**1P**(M118D)	MBP-Aes^N(M118D)-H_6	pCH92	pMAL-c2x	this work
**1P**(M118N)	MBP-Aes^N(M118N)-H_6	pCH93	pMAL-c2x	this work
**1P**(L119K)	MBP-Aes^N(L119K)-H_6	pCH94	pMAL-c2x	this work
**1P**(T69K/F75H)	MBP-Aes^N(T69K/F75H)-H_6	pCH138	pMAL-c2x	this work
**1P**(T69K/V84A)	MBP-Aes^N(T69K/V84A)-H_6	pCH137	pMAL-c2x	this work
**1P**(T69K/M118N)	MBP-Aes^N(T69K/M118N)-H_6	pCH100	pMAL-c2x	this work
**1P**(F75H/V84A)	MBP-Aes^N(F75H/V84A)-H_6	pCH134	pMAL-c2x	this work
**1P**(F75H/M118N)	MBP-Aes^N(F75H/M118N)-H_6	pCH136	pMAL-c2x	this work
**1P**(V84A/M118N)	MBP-Aes^N(V84A/M118N)-H_6	pCH135	pMAL-c2x	this work
**1P**(T69K/F75H/V84A)	MBP-Aes^N(T69K/F75H/V84A)-H_6	pCH143	pMAL-c2x	this work
**1P**(T69K/F75H/M118N) (**15P**)	MBP-Aes^N(T69K/F75H/M118N)-H_6 (MBP-YIDTD-CLm^N-H_6)	pCH145	pMAL-c2x	this work
**1P**(T69K/V84A/M118N)	MBP-Aes^N(T69K/V84A/M118N)-H_6	pCH142	pMAL-c2x	this work
**1P**(F75H/V84A/M118N)	MBP-Aes^N(F75H/V84A/M118N)-H_6	pCH144	pMAL-c2x	this work
**1P**(T69K/F75H/V84A/M118N)	MBP-Aes^N(T69K/F75H/V84A/M118N)-H_6	pCH424	pMAL-c2x	this work
**2P**	Aes^C-SVYLN-sfGFP (**Precursor**: H_6-smt3-Aes^C-SVYLN-sfGFP)	pCH196	pET28b	this work
**3P**	SBP-Aes^C-SVYLN-SBP	pTT22	pET16b	Ref[1]
**4P**	sfGFP-YIDTD-Aes^N-H_6	pCH84	pMAL-c2x	this work
**5P**	Aes^C(N159A)-SVYLN-sfGFP (**Precursor**: H_6-smt3-Aes^C(N159A)-SVYLN-sfGFP)	pCH188	pET28b	this work
**6P**	H_6-Smt3-DTD-(S1A)Aes^N	pCH148	pET28b	this work
**7P**	DTD-Aes^N (**Precursor**: H_6-Smt3-DTD-Aes^N)	pCH182	pET28b	this work
**8P**	DTD-(S1A)Aes^N (**Precursor**: H_6-Smt3-DTD-(S1A)Aes^N)	pCH148	pET28b	this work
**9P**	MYIDTD-(S1A)-Aes^N-GSH-Aes^C(N159A)-SVYLN (**Precursor**: MYIDTD-Aes^N(S1A)-GSH-Aes^C(N159A)-SVYLN-GyrA-CBD)	pZY49	pTWIN1	this work
**9P**(I7Q)	(S1A)-Aes^N(I7Q)-GSH-Aes^C(N159A)	pCH112	pTWIN1	this work
**9P**(T69K)	(S1A)-Aes^N(T69K)-GSH-Aes^C(N159A)	pCH39	pTWIN1	this work
**9P**(F75H)	(S1A)-Aes^N(F75H)-GSH-Aes^C(N159A)	pCH140	pTWIN1	this work
**9P**(V84A)	(S1A)-Aes^N(V84A)-GSH-Aes^C(N159A)	pCH139	pTWIN1	this work

Protein	Construct	Encoding Plasmid	Vector System	Reference
**9P**(M118N)	(S1A)-Aes^{N}(M118N)-GSH-Aes^{C}(N159A)	pCH151	pTWIN1	this work
**10P**	MDTD-(S1A)-Aes^{N}-GSH-Aes^{C}(N159A)-SVYLN (**Precursor**: MDTD-Aes^{N}(S1A)-GSH-Aes^{C}(N159A)-SVYLN-GyrA-CBD)	pCH271	pTWIN1	this work
**11P**	MBP-(S1A)Aes^{N1}(S1-N61)-H_6	pCH129	pMAL-c2x	this work
**12P**	MBP-(S1A)Aes^{N1}(S1-H68)-H_6	pCH118	pMAL-c2x	this work
**13P**	MBP-Aes^{N2}(I62-T120)-H_6	pCH130	pMAL-c2x	this work
**14P**	MBP-Aes^{N2}(T69-T120)-H_6	pCH117	pMAL-c2x	this work
**15P**	MBP-CLm^{N}-H_6	pCH145	pMAL-c2x	this work
**16P**	MYIDTD-(S1A)-CLm^{N}-GSH-Aes^{C}(N159A)-SVYLN (**Precursor**: MYIDTD-CLm^{N}(S1A)-GSH-Aes^{C}(N159A)-SVYLN-GyrA-CBD)	pCH163	pTWIN1	this work
**17P**	ALFAnb-CLm^{N}-H_6	pCH152	pET16b	this work
**18P**	ALFAnb-Aes^{N}-H_6	pCH222	pET16b	this work
**19P**	H_6-smt3-Aes^{C}-SVYLN-sfGFP	pCH196	pET28b	this work
	H_6-Smt3-DTD-Aes^{N}	pCH182	pET28b	this work
	EgA1nb-Aes^{N}-H_6	pCH223	pET16b	this work
**20P**	EgA1nb-CLm^{N}-H_6	pCH200	pET16b	this work
**21P**	H_6-Aes^{C}-ALFAnb-SBP	pCH204	pET16b	this work
**22P**	sfGFP-ALFAtag-H_6	pBJ278	pET22a	Ref[2]
**23P**	CAD-CLm^{N} (**Precursor**: H_6-smt3-CTD-Aes^{N})	pCH291	pET28b	this work
**24P**	H_6-Aes^{C}-ALFAnb(G47C)-SBP	pCH294	pET16b	this work
**25P**	MBP-Ssp^{N}-H_6	pTK56	pTrc99a	Ref[3]
**26P**	MBP-Ssp^{C}-H_6	pTK55	pMAL-c2x	Ref[3]
**27P**	Strep-eGFP-Npu^{N}	pVS07	pRSFDuet1	Ref[4]
**28P**	Npu^{C}-eGFP (**Precursor**: H_6-Smt3-Npu^{C}-eGFP)	pCH451	pET28b	this work

Supplementary Table 2 List of plasmids used for mammalian cell culture.

Plasmid	Construct	Encoding Plasmid	Vector System	Reference
**1**	Ig-kappa-HA-EGFR-mKoκ	pAA147	pDisplay	this work

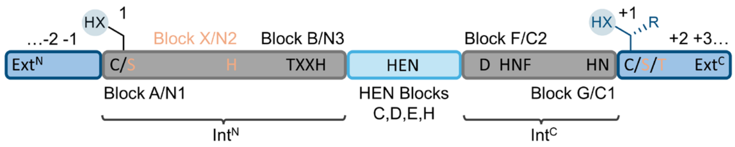

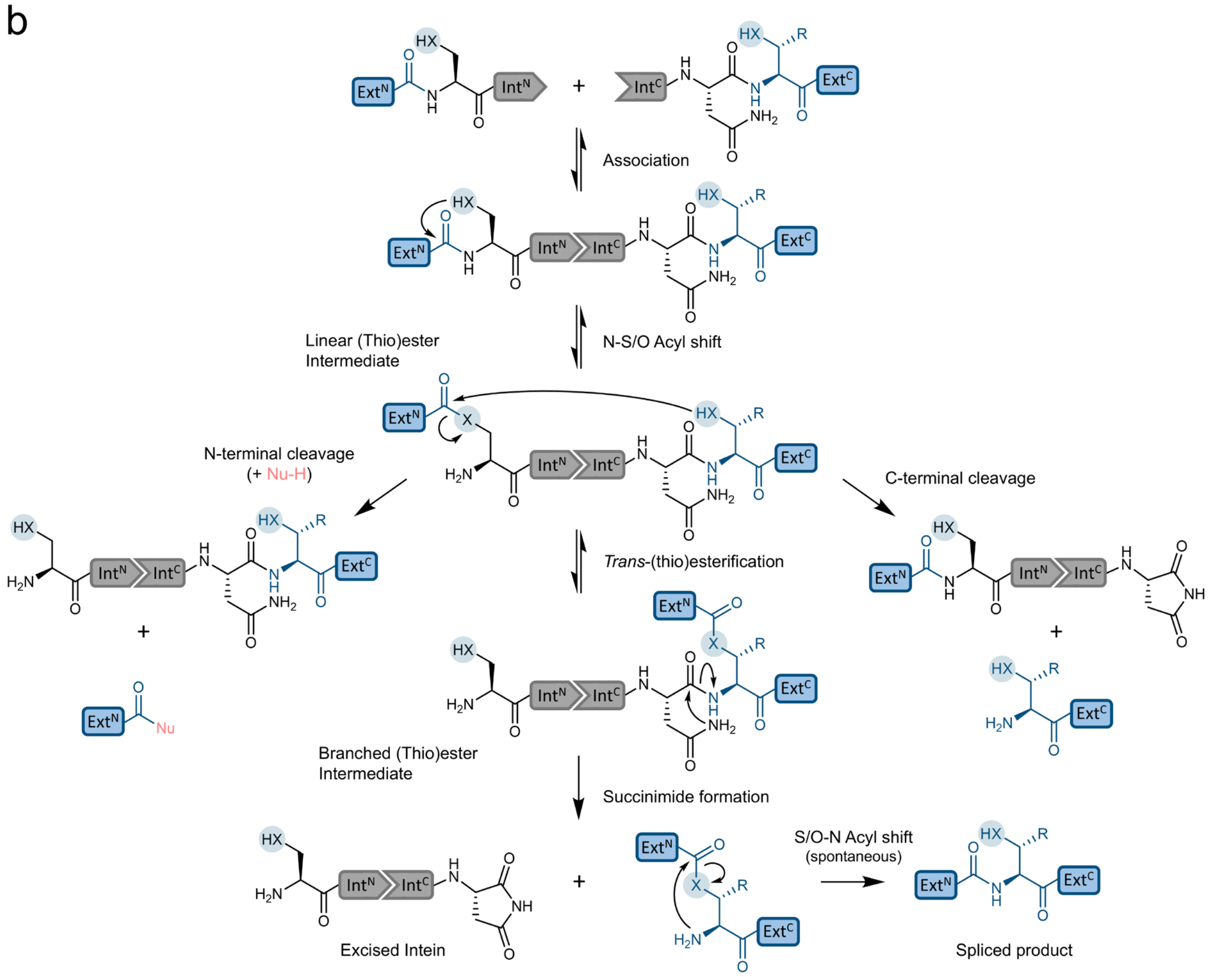

Supplementary Figure 1 Intein sequence features and protein splicing mechanism. **a** Conserved sequence motifs of class 1 inteins relative to the intervening homing endonuclease domain. **b** General protein splicing mechanism of split inteins (X = S, O; R = CH_3, H). Note that cysteine-less inteins operate with Ser1 and Ser+1 (Thr+1) residues at the two splice junctions to form the linear and branched ester intermediates. HEN: homing endonuclease domain.

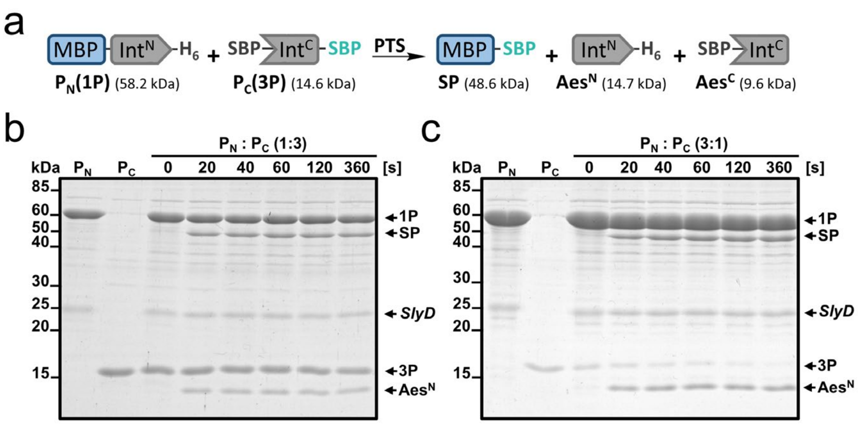

Supplementary Figure 2 The C-terminal precursor is completely consumed when the N-terminal precursor is given in excess (additional data to Fig. 1d). **a** Scheme of the PTS reaction. **b** SDS-PAGE analysis of the PTS reaction using **1P** with **3P** in three-fold molar excess (10 µM to 30 µM at 37°C). This experiment was repeated two times. **c** SDS-PAGE analysis of the PTS reaction using **1P** in three-fold molar excess to **3P** (30 µM to 10 µM at 37°C). SlyD is indicated as a known contamination of Ni-NTA-purified proteins.[5] This experiment was repeated two times.

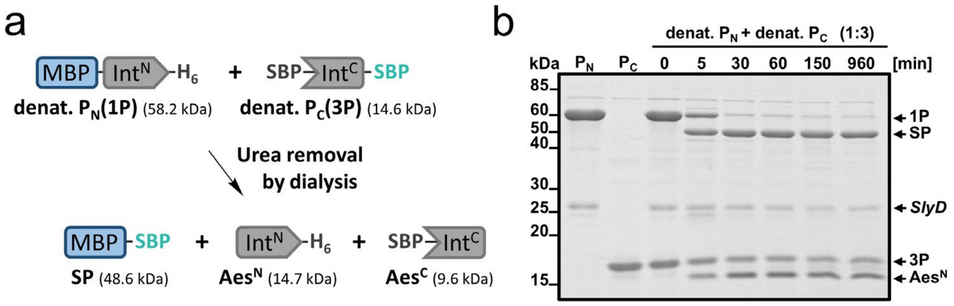

Supplementary Figure 3 Collective refolding of both split intein precursor proteins leads to quantitative splicing. **a** Scheme of the experiment. Individual precursor proteins were under denaturing conditions with 8 M urea. After mixing, the urea was removed by dialysis and spontaneous protein *trans*-splicing was observed. **b** SDS-PAGE analysis of the reaction using denatured **1P** and denatured **3P** at 10 µM and 30 µM, respectively. The reaction was performed at 25°C. Aliquots were removed during the dialysis at the indicated time points, quenched with SDS sample buffer and analyzed by SDS-PAGE. Note that the N-terminal precursor **1P** is nearly completely consumed. A contaminating band was identified as SlyD as indicated.[5] Shown is a gel stained with Coomassie brilliant blue.

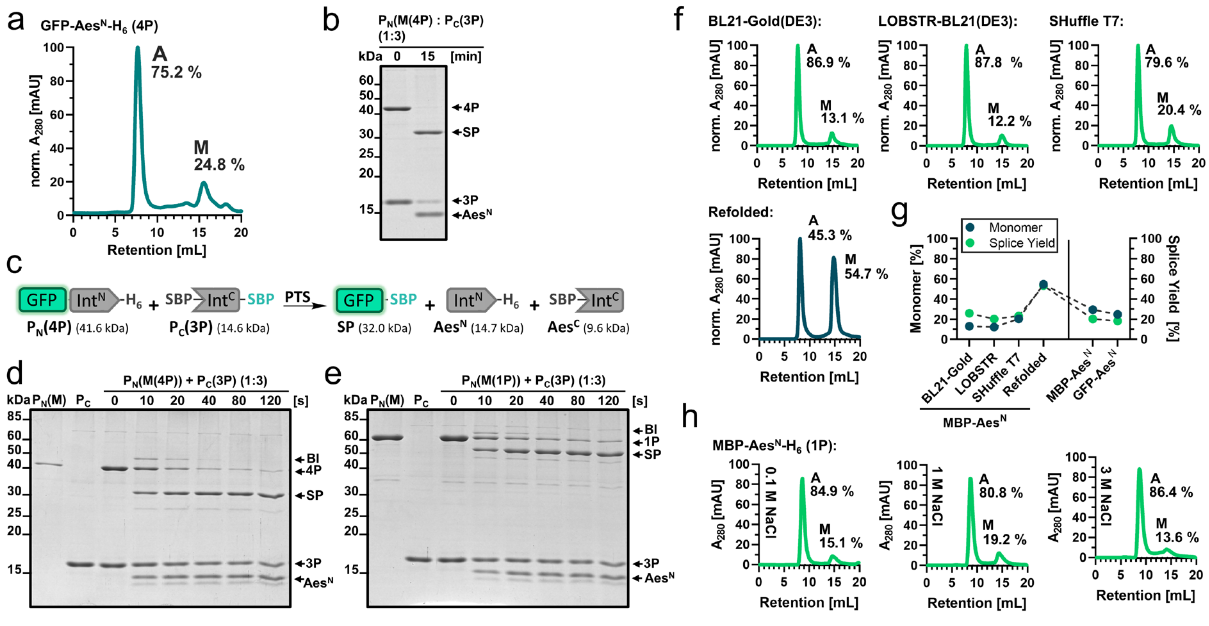

Supplementary Figure 4 Aes^N fragment aggregation is independent of the extein context, the expression system and ionic strength (additional data to Fig. 3). **a** Normalized UV elution profile of **4P** separated into two fractions on a Superdex 200 Increase 10/300 GL prepacked column (GE Healthcare) at a flow rate of 0.5 mL/min. **b** SDS-PAGE of the PTS reaction using the monomeric species of **4P** (10 µM) with three-fold excess of **3P** (30 µM) at 37°C. Shown is a gel stained with Coomassie brilliant blue. **c** Scheme of the PTS reaction using construct **4P** and **3P** (SBP-Aes^C-SBP) as performed in panel D and quantified in Figure 2D. **d** SDS-PAGE analysis of the time-resolved PTS reaction using the monomeric species of **4P** (10 µM) (purified with the monomer isolation protocol) with three-fold excess of **3P** (30 µM) at 37°C. This experiment was repeated two times. **e** SDS-PAGE analysis of the time-resolved PTS reaction using the monomeric species of **1P** (10 µM) (purified with the monomer isolation protocol) with three-fold excess of **3P** (30 µM) at 37°C. This experiment was repeated two times. **f** SEC-chromatograms of different **1P** samples (all at 1.5 mg/mL) purified by Ni-NTA chromatography, but produced by using different *E. coli* expression systems or an additional step-wise refolding step as indicated. SEC was performed on a Superdex 200 Increase 10/300 GL prepacked column (GE Healthcare) at a flow rate of 0.75 mL/min. **g** Correlation between the proportion of monomer and splice product formation. Shown are the monomer proportion after recombinant Aes^N precursor production in different expression systems and with different N-exteins compared to the final splice yield reached with these precursors. The monomer proportion (blue) of **1P** and **4P** was analyzed by SEC using a Superdex 200 Increase 10/300 GL prepacked column (GE Healthcare) after incubation at 10 µM and 15°C for 24h. The splice efficiency (green) was analyzed by SDS-PAGE using **3P** in three-fold molar excess (10 µM to 30 µM, 37°C) to the Aes^N precursor after 1 h incubation. **h** SEC-chromatograms of different samples of **1P** (1 mg/mL) after incubation with the indicated salt concentrations for 12 h. SEC was performed on a Superdex 200 Increase 10/300 GL prepacked column (GE Healthcare) at a flow rate of 0.75 mL/min. Source data are provided as a Source Data file.

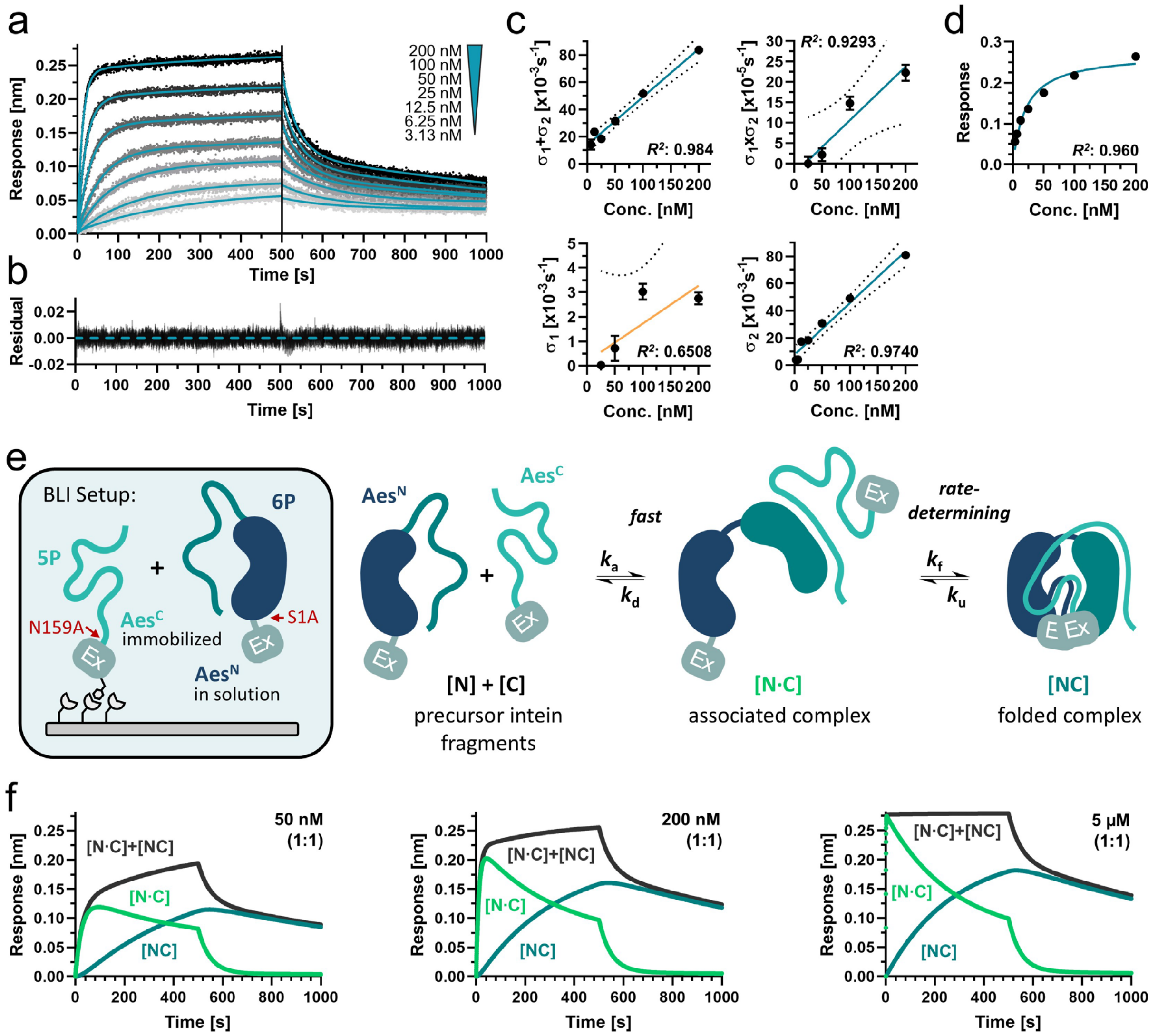

Supplementary Figure 5 Investigation of the Aes split intein assembly mechanism by biolayer interferometry (BLI). **a** BLI sensorgram using biotinylated Aes^C-GFP(N159A) (**5P**) as immobilized ligand on the sensor tip and smt3-(S1A)Aes^N (**6P**) as the analyte at different concentrations. The association and dissociation profiles were fitted by double exponential functions. **b** Residual plot of the BLI profiles to the applied fit. **c** Steady state analysis of the BLI response at 490 s – 500 s plotted against the analyte concentration. The upper graph shows the plot with a linear scale of the analyte concentration while for the lower graph a logarithmic scale was used. **d** Sum of σ_1+σ_2 from the double exponential function plotted against the concentration. The continuous line represents a linear fit. Product of $\sigma_1\sigma_2$ plotted against the concentration, σ_1 plotted against the concentration and σ_2 plotted against the concentration (details in the method section). **e** Scheme of the experimental BLI setup and the underlying intein assembly mechanism. **f** Simulation of the underlying binding kinetics of [N·C] and [NC] using the determined binding parameters at different concentrations. Note, that the sum [N·C] + [NC] resembles the BLI output. For panel **a** and **d**, n = 7 technical replicates were used measured at different concentrations as indicated. Data are presented normalized to a control without analyte protein **6P**. For panel **c**, data and s.d. are derived from the fit shown in panel **a** and presented as mean ± s.d.. Source data are provided as a Source Data file.

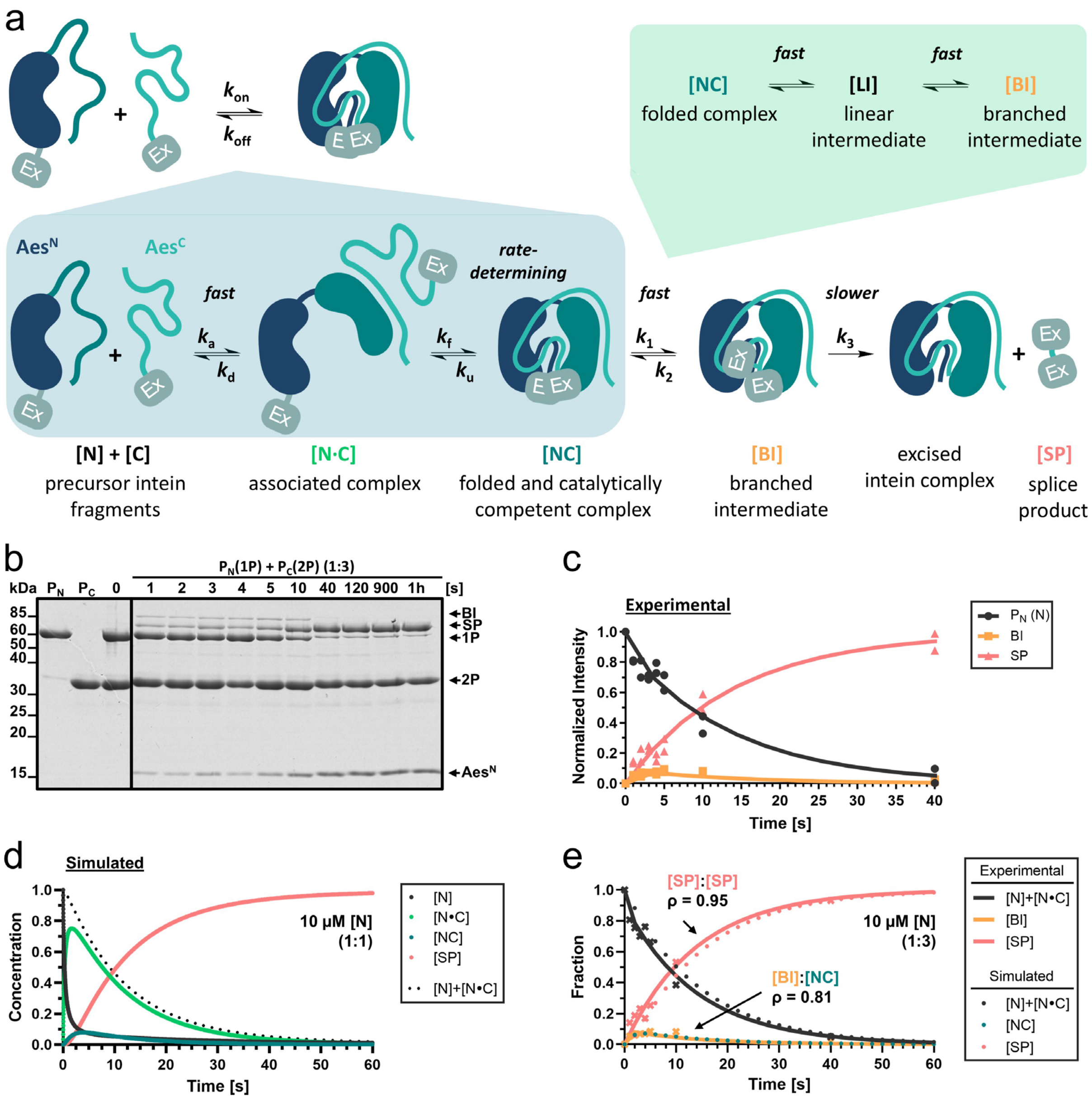

Supplementary Figure 6 Investigation of the underlying relationship between the Aes assembly and splice mechanism. **a** Scheme of the underlying assembly and simplified thee-state kinetic splicing mechanism. **b** SDS-PAGE analysis of the PTS reaction using monomeric **1P** and **2P** with a three-fold molar excess of **2P** (10 µM **1P** to 30 µM **2P**) at 37°C. This experiment was repeated two times. **c** Time course of the PTS reaction under pseudo-unimolecular conditions fitted to a three-step kinetic model showing the formation and resolution of the branched intermediate. Only the active fraction (80%) of the precursor protein P_N was considered to determine the formation of the branched intermediate and the splice product. **d** Simulation of the underlying binding and splice kinetics over a time course of 60 s with Aes^{N} and Aes^{C} applied in equimolar concentrations (10 µM). **e** Correlation between the simulated binding kinetics and splice parameters and the experimentally determined BI and SP formation using Aes^{C} in three-fold excess (10 µM Aes^{N} to 30 µM Aes^{C}). Note, that the measured accumulation of the brached intermediate (BI) resembles the simulated time course of the folded complex [NC]. For panel **e**, *n* = 2 technical replicates, *p*-values were derived from a two-tailed non-parametric Spearman correlation test. Source data are provided as a Source Data file.

Supplementary Table 3 Kinetic binding parameters of the Aes123 PolB1 intein.*

**Binding Parameter**		**Determined with inactive intein**	**Simulated for active intein**
association rate	k_a	$3.53 \pm 0.23 \times 10^5$ $M^{-1}s^{-1}$	
dissociation rate	k_d	$1.76 \pm 0.13 \times 10^{-2}$ s^{-1}	
folding rate	k_f	$2.84 \pm 1.02 \times 10^{-3}$ s^{-1}	$91.13 \pm 6.17 \times 10^{-3}$ s^{-1}
unfolding rate	k_u	$8.73 \pm 0.90 \times 10^{-4}$ s^{-1}	
overall ass. rate	k_{on}	$4.91 \pm 1.83 \times 10^4$ $M^{-1}s^{-1}$	$29.59 \pm 3.27 \times 10^4$ $M^{-1}s^{-1}$
overall diss. rate	k_{off}	$7.51 \pm 1.11 \times 10^{-4}$ s^{-1}	$7.51 \pm 0.32 \times 10^{-4}$ s^{-1}
dissociation constant	K_d	11.7 ± 3.0 M^{-9}	0.48 ± 0.12 M^{-9}

**Splice Kinetics**		**Determined**	**Used in Simulation**
overall splice rate	k_{total}	$91.13 \pm 6.17 \times 10^{-3}$ s^{-1}	$91.13 \pm 6.17 \times 10^{-3}$ s^{-1}
succinimide resolution	k_3	$0.78 \pm 0.13 \times 10^{-3}$ s^{-1}	
splice rate	k_{splice}		$0.78 \pm 0.13 \times 10^{-3}$ s^{-1}

***** Experimentally determined binding parameters by biolayer interferometry using splice inactive constructs and simulated values for the active Aes intein. To consider the influence of active protein *trans*-splicing on the binding parameters, the splice kinetics determined with the monomeric construct **1P** and **3P** (10 µM to 30 µM, k_{total} = 91.13 ± 6.17 x 10^{-3} s^{-1}) and monomeric **1P** and **2P** (5 µM to 15 µM, 70.04 ± 5.45 x 10^{-3} s^{-1}) were used.

Supplementary Note 1 Biophysical and kinetical investigation of the underlying assembly mechanism by biolayer interferometry.

We investigated the underlying binding kinetics of the Aes123 PolB1 intein by biolayer interferometry using biotinylated Aes^{C}(N39A)-GFP (**5P**) as immobilized ligand applied to different concentrations of Smt3-(S1A)Aes^{N} (**6P**) as analyte (Supplementary Fig. 5a, b, e). The BLI profiles revealed a biphasic binding behavior which suggests a similar multistep assembly mechanism as described for other split inteins.[6] To determine the binding parameters, we fitted the association and dissociation profiles by double exponential functions and extracted the rate constants by an analytical solution of the biphasic rate equations. Here, the dependencies of the exponents and their sums and products on the analyte concentration (C) were used to confirm the correct model and to determine the rate equations as described by Tiwari *et al.*.[7] The linearity between $\sigma_1 + \sigma_2$ and C verifies that the underlying binding mechanism reflects a linear biphasic reaction. Moreover, the linear relationship of the product $\sigma_1\sigma_2$ to C indicates a binding mechanism as depicted by the two-step conformational change model and confirms the proposed intein assembly mechanism (Supplementary Fig. 5c). The concentration dependent association rate (k_a) was obtained from the slope of the sum of the exponents plotted against the concentration with k_a = 3.53 ± 0.23 x 10^5 $M^{-1}s^{-1}$. Based on the two-step conformational change model we calculated a K_D value of 11.7 ± 3.0 nM with an overall k_{on} of 4.91 ± 1.83 x 10^4 $M^{-1}s^{-1}$ and a k_{off} of 7.51 ± 1.11 x 10^{-4} s^{-1} (Supplementary Table 3). This high affinity in the low nanomolar range is a typical feature of native split inteins and confirmed by the steady state analysis which assumes a similar K_D value (14 nM) (Supplementary Fig. 5d). Consequently, we simulated binding curves at different concentrations together with the obtained rate constants of the dissociation rate (k_d = 1.76 ± 0.13 x 10^{-2} s^{-1}), the folding rate (k_f = 2.84 ± 1.02 x 10^{-3} s^{-1}) and the unfolding rate (k_u = 8.73 ± 0.90 x 10^{-4} s^{-1}). The simulations resemble the experimental BLI data and clearly identify the folding step as rate determining (Supplementary Fig. 5f).

However, it has to be considered that the affinity measurements were done with the inactivated constructs which probably lead to an underestimation of the real binding kinetics since the equilibrium between folding and unfolding is influenced by the splice rate (k_{splice}) (Supplementary Fig. 6a). In a multistep mechanism the rate constants of the rate limiting step defines the overall rate constants. However, the determined folding rate (k_f) is slower than the initially obtained overall splice rate (k_{total}). We simulated the real binding kinetics at equimolar concentrations (10 µM) by equating the folding rate constant with the previously determined overall splice rate of the PTS reaction ($k_{total} = k_f$). Moreover, a highly time-resolved splice assay was used to determine the single catalytic steps simplified by a three-state kinetic model (Supplementary Fig. 6b, c). We were not able to exactly define k_1 and k_2 due to the fast reaction rate and low accumulation of the branched intermediate (BI), but we could extract the BI resolution as rate determining step within the splice mechanism with k_3 = 0.78 ± 0.13 x 10^{-3} s^{-1}.

Interestingly, equating k_3 as k_{splice} simulates binding and splice kinetics that assume overall splice rates which lie in the realm of the experimentally determined values (Supplementary Fig. 6d). The extrapolated kinetic parameters for the active intein fragments (Supplementary Table 3) suggest a K_D of 0.48 ± 0.12 nM and the corresponding simulated splice product formation even slightly underestimates the experimentally measured product formation (Supplementary Fig. 6e). However, both curves still show a good Spearman correlation coefficient with $\rho = 0.95$ ($p = 0.00035$). Moreover, the simulated appearance of the folded complex strongly adopts the time-resolved accumulation of the branched intermediate with a correlation of $\rho = 0.81$ ($p = 0.011$). This correlation corroborates the fast transesterification into the BI upon fragment folding. This fast conversion (k_1) in combination with a fast resolution of the branched oxo-ester intermediate (k_2) explains the low BI accumulation and the difficulty in determining these parameters.

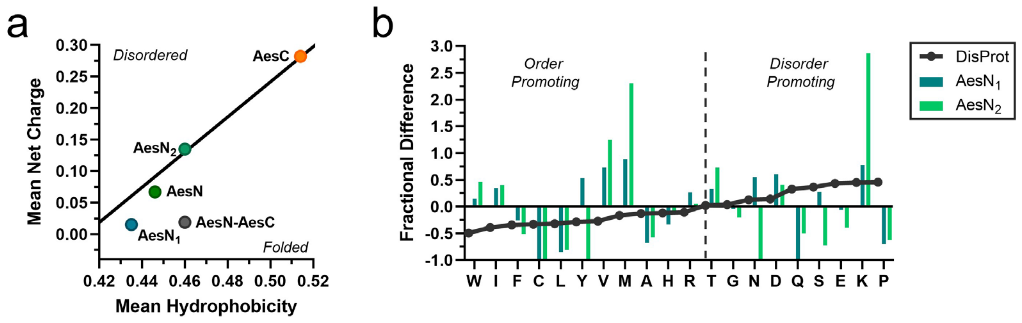

Supplementary Figure 7 Sequence-based prediction of the folding state by charge-hydrophobicity plot and amino acid composition profiling. **a** Charge-hydrophobicity (CH) plot of the Aes^N fragment, the Aes^C fragment, the N-terminal lobe of Aes^N ($AesN_1$, aa1-68), the C-terminal lobe of Aes^N ($AesN_2$, aa69-120) and the fused complex structure (Aes^N-Aes^C). A linear boundary (R = 2.785 H −1.151) separates the CH plot into disordered (above) and ordered (below) regions. **b** Comparison of amino acid composition of $AesN_1$, $AesN_2$ and the Disprot 9.6 database (representing known disordered regions) with the general protein database UniProtKB/TrEMBL 2024_04. The bar graph represents the amino acid enrichment (positive) or depletion (negative) relative to TrEMBL. The $AesN_1$ and $AesN_2$ amino acid composition in **b** does not show the typical distribution as known for intrinsically disordered proteins.[8] Rather the amino acid distribution reflects order promoting as well as disorder promoting characteristics. These sequence characteristics accompany the known/putative intein assembly mechanism which requires the same amino acid sequence in disordered and ordered states at different assembly steps. Notably, the overrepresentation of the charged lysine residue in $AesN_2$ indicates a central role in holding the intein fragment structure partially disordered. Note that a high net charge in the $IntN_2$ lobe is conserved among inteins.[6] Source data are provided as a Source Data file.

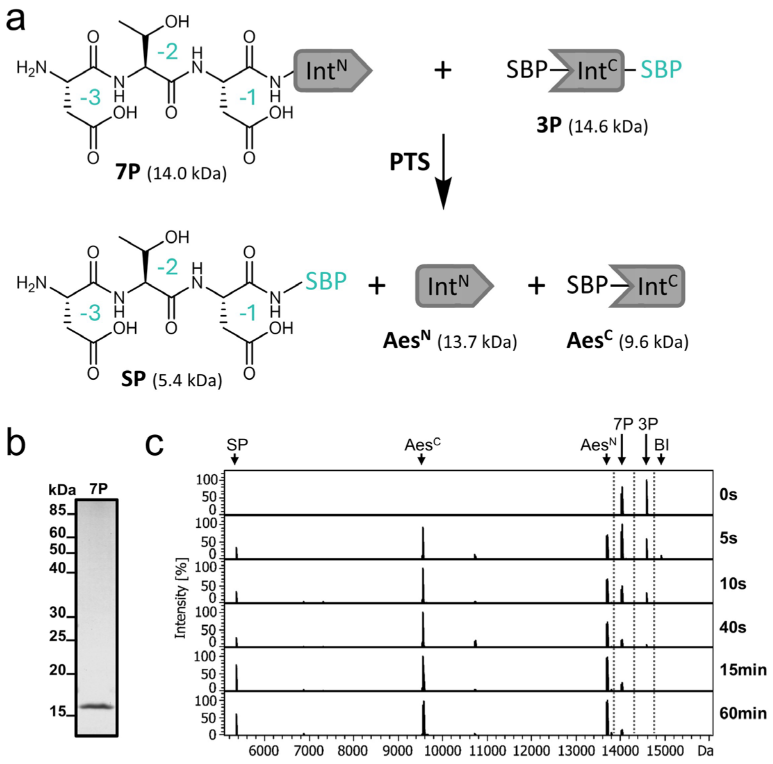

Supplementary Figure 8 Splice activity of the Aes^N precursor with a short N-terminal extein of only 3 residues. **a** Scheme of the PTS reaction. **b** SDS-PAGE analysis of purified monomeric DTD-Aes^N (**7P**) (prepared by the monomer isolation protocol). **c** ESI-MS analysis of the PTS reaction using the monomeric species of **7P** in low excess to **3P** (15 µM to 10 µM) at 37°C.

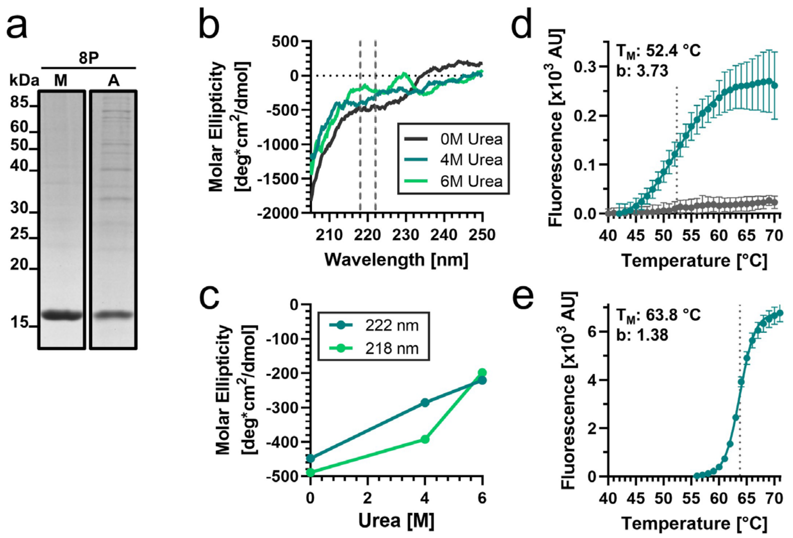

Supplementary Figure 9 Biophysical investigation of the Aes^{N} precursor (additional data to Fig. 4a). **a** SDS-PAGE analysis of the purified monomeric (prepared by the monomer isolation protocol) and aggregated species of DTD-Aes^{N}(S1A) (**8P**) used for the biophysical analysis. **b** Far UV circular dichroism spectroscopy of 10 µM **8P** containing different concentrations of urea as indicated. **c** Molar ellipticity of **8P** at 218 nm and 222 nm as shown in **b** plotted against urea concentration. **d** Thermal shift assay of DTD-Aes^{N}(S1A) (**8P**; 0.2 g/L) shown next to a buffer control (gray). The data was fitted to a sigmoidal four-parameter logistic equation to determine the melting temperature (T_M) and the folding cooperativity (b; Hill coefficient (no unit)). To this end, the data profile was cut at the highest value (at 70°C) to account for post-peak aggregation of protein-dye complexes leading to quenching of the fluorescence signal. **e** Thermal shift assay of MYIDTD-Aes^{N}(S1A)-GSH-Aes^{C}(N159A)-SVYLN (**9P**, 0.5 g/L) fitted to a sigmoidal four-parameter logistic equation. For panel **d** and **e**, *n* = 7 and 5 technical replicates, respectively. Data are presented as mean ± s.d. normalized to the highest fluorescence intensity. Source data are provided as a Source Data file.

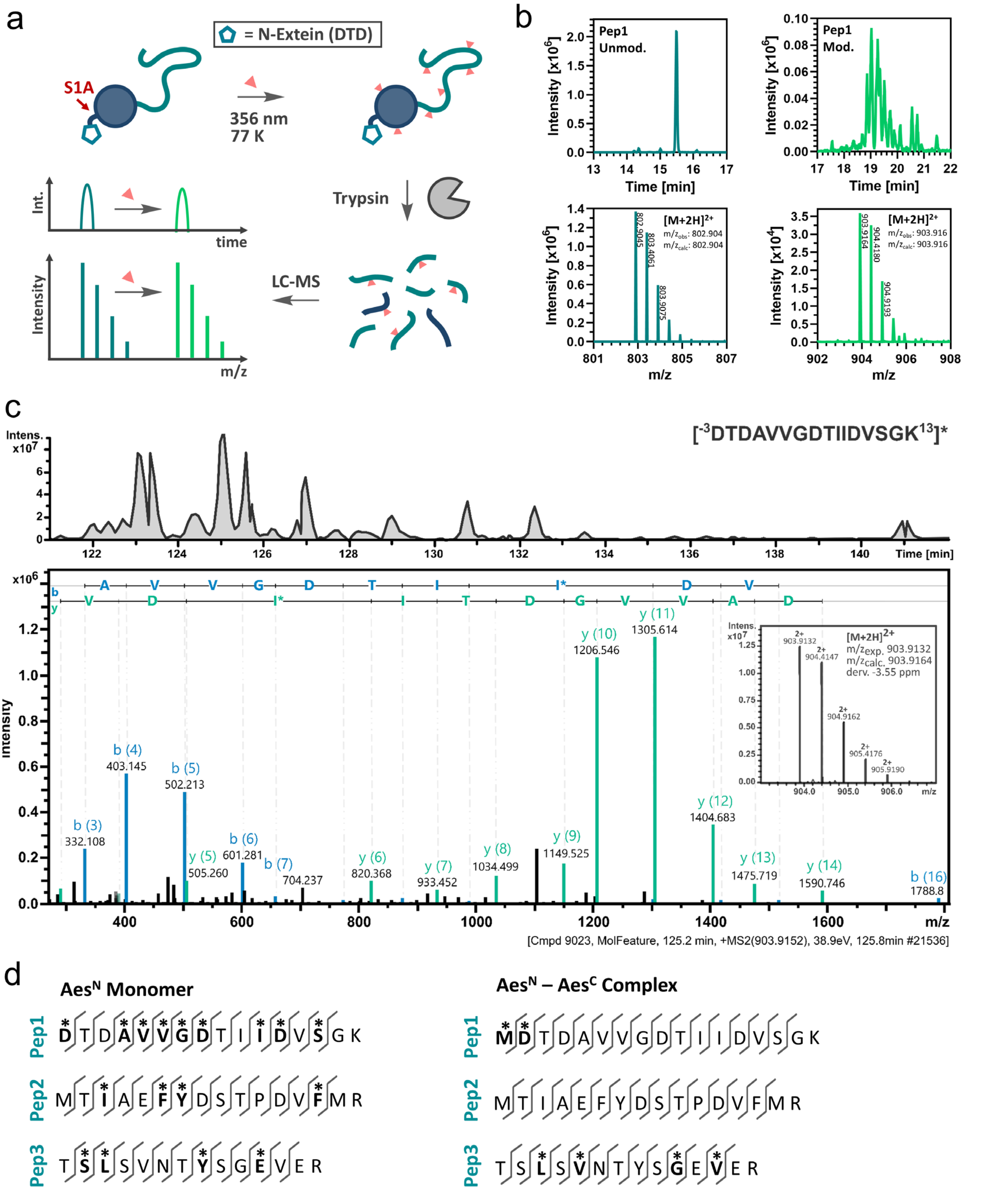

Supplementary Figure 10 Principle of the photoreactive carbene labeling and LC-MS based footprinting (additional data to Fig. 4c). **a** Scheme of the experimental workflow. **b** Representative extracted ion chromatogram (EIC) after protein trypsinization of unlabeled **Pep1** (blue) and carbene-labeled **Pep1** (green) of the carbene footprinting approach using 10 µM DTD-Aes^{N}(S1A) (**8P**). The upper panels show the EIC of unlabeled **Pep1** (blue) and carbene-labeled **Pep1** (green). The lower panels show the MS spectra of unlabeled **Pep1** (blue) and carbene-labeled **Pep1** (green). **c** LC-MS/MS analysis of the carbene-modified and trypsinated peptides. Shown are representative samples of the non-quantitative tandem MS analysis of carbene-labeled **Pep1.** The upper panel shows the EIC of carbene-labeled **Pep1**, containing various singly labeled species with different retention times. The lower panel shows the MS^{2} spectrum of one such **Pep1** species, which revealed that the carbene-label was inserted at position I8. The inset shows the MS^{1} spectrum of carbene-modified **Pep1**. **d** Summary of the identified carbene modification sites by the non-quantitative tandem MS analysis of **Pep1**, **Pep2** and **Pep3** compared between the monomeric Aes^{N} precursor DTD-Aes^{N}(S1A) (**8P**) and the Aes^{N}-Aes^{C} complex structure of MDTD-Aes^{N}(S1A)-GSH-Aes^{C}(N159A)-SVYLN (**10P**). Indicated are amino acids identified as carbene labeled (asterisk).

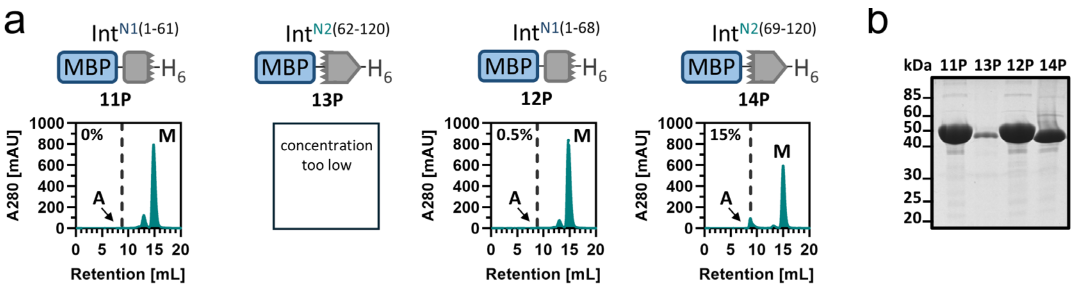

Supplementary Figure 11 SEC analysis of the single Aes^{N} fragment segments fused to MBP (additional data to Fig. 4d). **a** SEC-analysis with 150 µM of each indicated protein. The construct **13P** was not tested at these high concentrations due to lower solubility. **b** SDS-PAGE analysis of purified proteins after recombinant protein expression and Ni-NTA affinity chromatography. Note that the proteins were produced under the same expression and purification conditions, leading to different protein yields. Source data are provided as a Source Data file.

Supplementary Table 4 Crystallographic data collection and structure refinement.*

**Data Collection**	PDB ID: 9HTH
Space Group	$P\ 2_1\ 2_1\ 2_1$
Unit-cell parameters	
a, b, c (Å)	30.1, 63.0, 162.4
α, β, γ (°)	90, 90, 90
Resolution range (Å)	34.13 - 1.38 (1.43 - 1.38)
Observed reflections	827722 (83755)
Unique reflections	64820 (5518)
Completeness (%)	96.27 (85.92)
Multiplicity	12.8 (13.1)
I/σ(I)	16.43 (1.73)
$CC_{1/2}$	0.999 (0.782)
Willson B-factor	19.07
R_{meas}	0.077 (1.235)
**Refinement**	
R_{work}, R_{free}	0.195 / 0.228
Reflections	62612
Ramachandran favored (%)	98.53
Ramachandran allowed (%)	1.47
Ramachandran outliers (%)	0
RMS bonds (Å)	0.007
RMS angles (°)	1.27
Average B-factor	27.16
macromolecules	26.7
solvent	33.7
Number of TLS groups	15

* Values in parentheses refer to the highest-resolution shell.

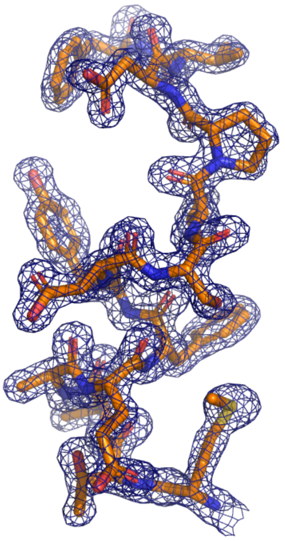

Supplementary Figure 12 Portion of the electron density map of the Aes intein crystal structure. Shown is the representative electron density of helix M15-F28 at 1.5 sigma contour level (map type 2Fo-Fc).

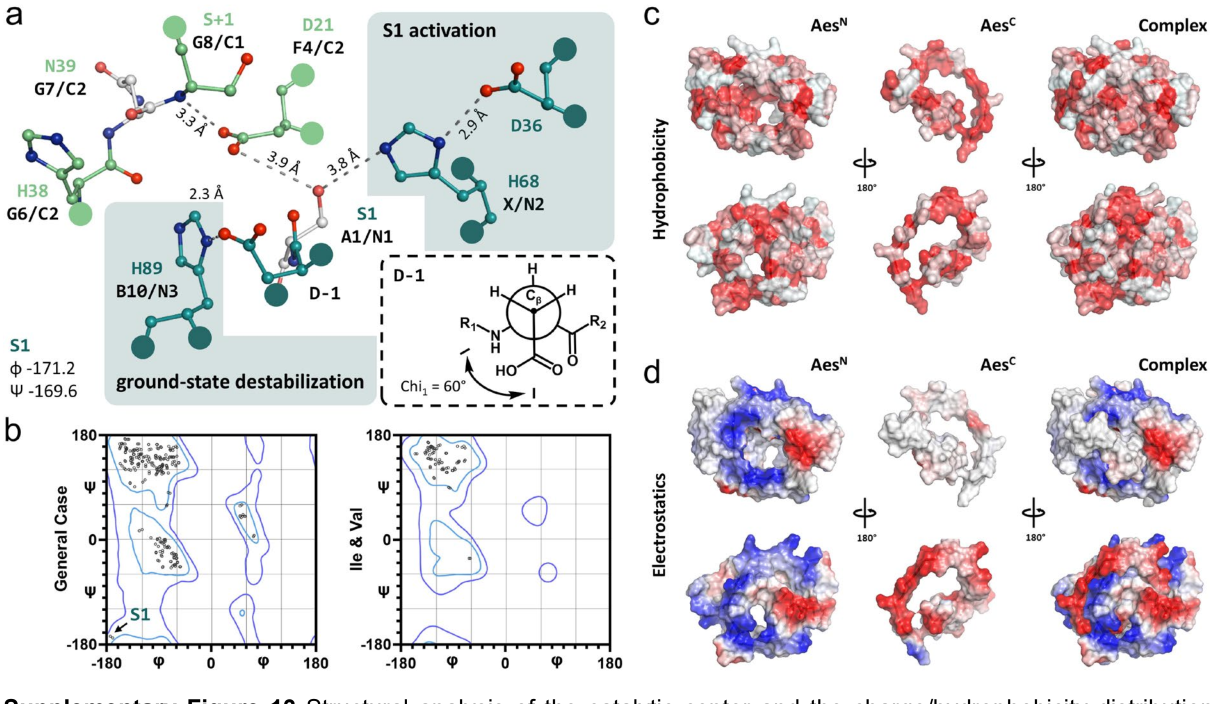

Supplementary Figure 13 Structural analysis of the catalytic center and the charge/hydrophobicity distribution among the intein fragments using the crystal structure of the Aes123 PolB1 intein. **a** Essential catalytic residues within the active site of the Aes123 PolB1 intein with the residues of the Aes^N fragment marked in blue and the Aes^C fragment labeled in green. Indicated are the catalytic residues and the corresponding conserved motifs as well as the distance between selected functional groups specified in (Å). The side chains of Ser1 and Asn159 (gray) are remodeled in the shown structure using Pymol as the intein was crystalized with S1A and N159A mutations. The Newman projection illustrates the energetically unfavorable gauche(-) conformation of the Asp-1 side chain. The conformation is defined by an angle of 60° between the Cγ and the amide nitrogen (R_1: N-terminal towards Thr-2; R_2: C-terminal with the scissile amide bond towards Ser1). **b** Ramachandran plots of the Aes123 PolB1 complex structure generated using MolProbity[9] highlighting the conformational constraints on the S1 backbone. The plot on the left-hand side treats all residues except Val, Ile, Pro and Gly. The plot on the right-hand side treats Val and Ile residues. **c** Surface view of the Aes123 PolB1 intein complex structure and the individual Aes^N and Aes^C fragments showing the surface hydrophobicity in red scale. **d** Surface view showing the electrostatic properties in red scale (negatively charged) and blue scale (positively charged).

Supplementary Note 2 Crystal structure of the Aes123 PolB1 intein.

We obtained crystals of the inactivated and fused Aes123 PolB1 precursor construct with 5 extein residues on each flank (construct **9P**) that diffracted to 1.38 Å resolution (Fig. 5c). The asymmetric crystal unit contained two molecules that exhibited only slight differences in electron density. Most notably, while in chain A none of the N-terminal and only three of the C-terminal extein residues were resolved, in chain B four and five residues, respectively, were visible. Overall, the intein adopted the typical horseshoe fold. The artificial GSH-linker between the Aes^N and Aes^C parts was well resolved. Interestingly, the region around residues ~25 to 40 (β3 and β4) showed high structural similarity to the insertion first found at the corresponding region in thermophilic inteins,[10] and later also in the mesophilic cysteine-less PolB16 OarG intein[11] (Supplementary Fig. 13a). Characteristically, the insertion folds into two additional β-sheets that extend the β7-strand at residues Y64-T69 (Fig. 5c, d). The preceding α-helix (α1) is disrupted by P25 and shorter than typically observed in the thermophilic counterparts and the PolB16 OarG intein.

The Aes123 PolB1 intein also contains the recently discovered conserved histidine that represents a signature sequence of cysteine-independent inteins in the newly defined motif NX (Supplementary Fig. 13b).[11] This residue, His68, is positioned close to the side chain of Ser1 (when remodeled back into the crystallized intein with the S1A mutation), thereby further supporting its proposed role in activation of the Ser1 side chain (Supplementary Fig. 12a). Interestingly, together with Asp37 a potential seryl-histidyl-aspartate motif is formed, the most common catalytic triad known from enzymatic studies.[12] This catalytic motif is unique to the Aes123 PolB1 intein within the known intein structures and might explain the high reaction rate of this cysteine-independent intein.

Another notable observation from the crystal structure is an unusual conformation of the first N-terminal extein amino acid. This residue, Asp-1, adopts an allowed but energetically unfavorable gauche-(g-) conformation, defined by an angle of + 60° between Cγ and the amide nitrogen (Supplementary Fig. 12a). The g- conformation appears to be stabilized by a hydrogen-bonding contact between the side chain carboxyl group with the π-nitrogen of the catalytic motif B/N3 histidine His89 (distance of 2.7 Å in chain B). This conformation indicates a functional role of His89 in the distortion of the upstream scissile bond to help facilitate the initial N-O acyl shift. The crystal structure gives hints to slight conformational strains on the scissile peptide bond with torsion angles of φ = -171.2 and ψ = -169.6 in chain B (Supplementary Fig. 12b).

With respect to the interaction of the Int^N with the Int^C fragment, the crystal structure revealed the expected regions of electrostatic complementarity in the folded intein complex while hydrophobic residues are rather uniformly distributed in structure and sequence (Supplementary Fig. 12c, d). The electrostatic complementarity is mostly localized in the intermolecular β-sheet composed of β7 (Aes^N) and β13 (Aes^C) and was consciously retained during the engineering process. Further, the structural integrity of the folded intein complex and the residue's physicochemical properties were considered for the engineering-based amino acid substitutions using the structural X-ray data.

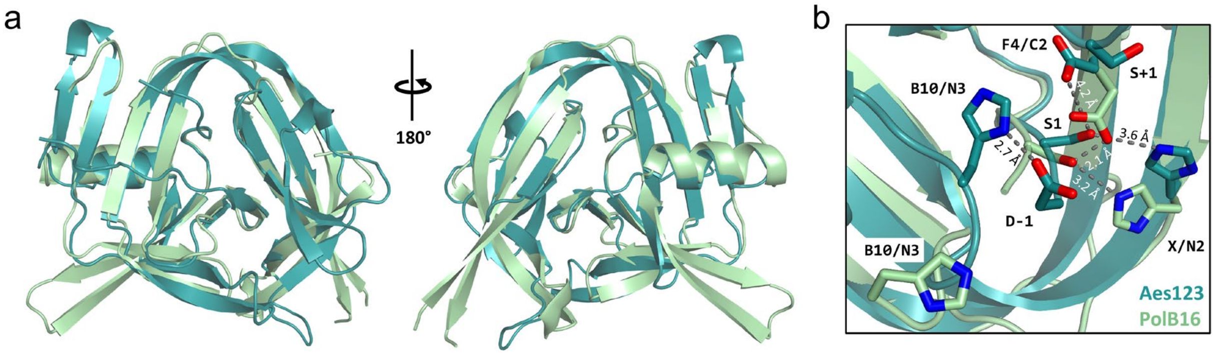

Supplementary Figure 14 Structural comparison between the cysteine-less Aes123 PolB1 and the PolB16 inteins (PDB ID: 8CPN).[11] **a** Overall structural alignment between both inteins. **b** Structural alignment of the catalytic center of both inteins, highlighting the previously described unusual position of the catalytic B10/N3 histidine in the PolB16 intein (which has to be seen in context with the following amino acid residues that were found disordered in the PolB16 crystal structure),[11] and the previously discovered catalytic block X (N2) histidine, which is conserved among cysteine-independent inteins.[11] Note that Ser1 side chains were modeled into the structures using PyMol, as the double mutants S1A and N159A (N183A for the PolB16 intein) were used for crystallization.

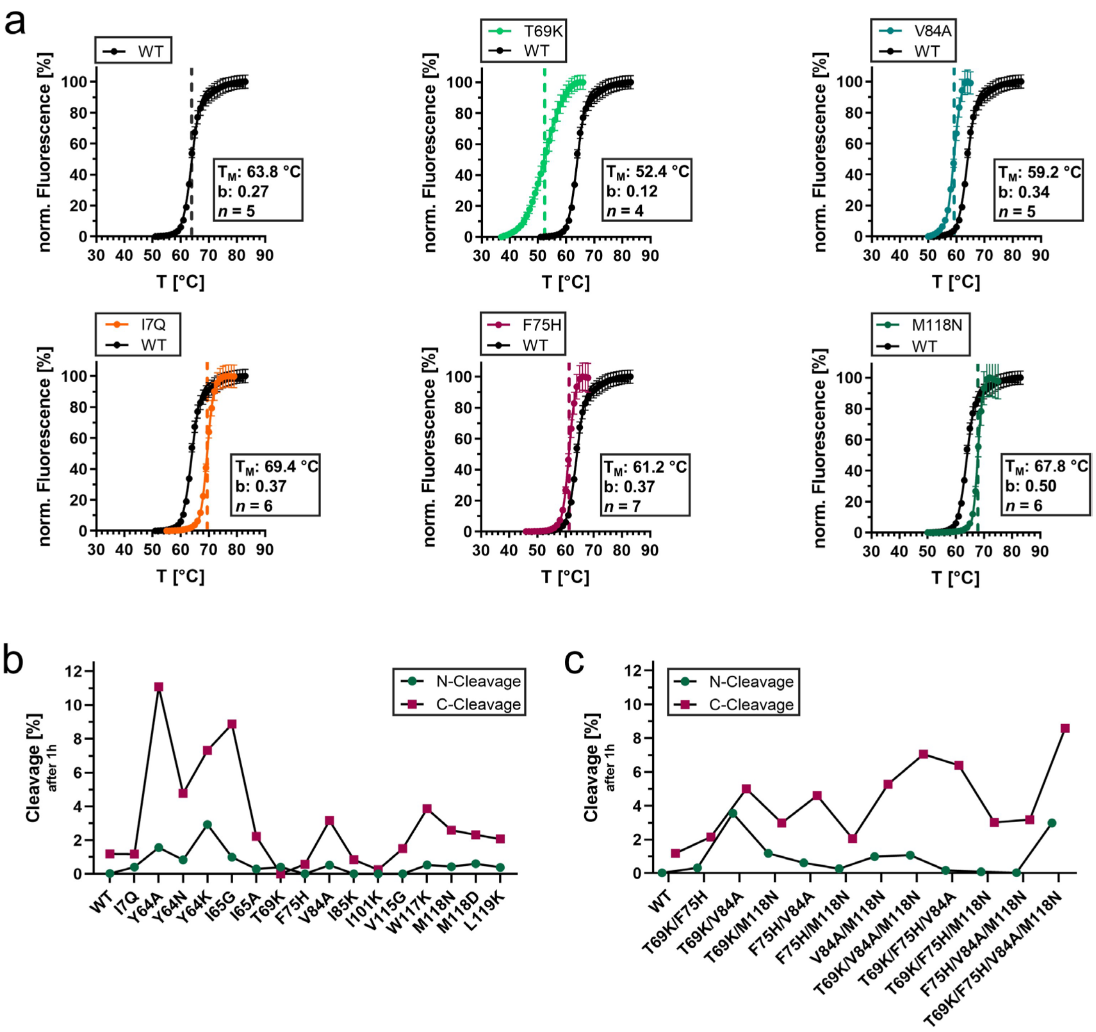

Supplementary Figure 15 Influence of the aggregation-reducing mutations on the extent of side reactions and on the folding cooperativity to the canonical intein complex structure. **a** Thermal stability of the indicated, selected single mutants compared to the parent Aes^N(S1A)-Aes^C(N159A) (WT; protein **9P**) fused complex structure. The denaturation profiles were fitted to a sigmoidal four-parameter logistic equation to determine the indicated melting temperatures (T_M) and the Hill coefficient (b) of the fit to investigate the cooperativity in defolding. **b** N- and C-cleavage of the single mutants in the PTS reaction after 1 h (P_N and P_C precursors were used at 5 and 15 µM concentration, respectively). **c** N- and C-cleavage of the double, triple and quadruple mutants in the PTS reaction after 1h. For panel **a**, *n* is specified in the figure. Data are presented as mean ± s.d. normalized to the highest fluorescence intensity. Source data are provided as a Source Data file.

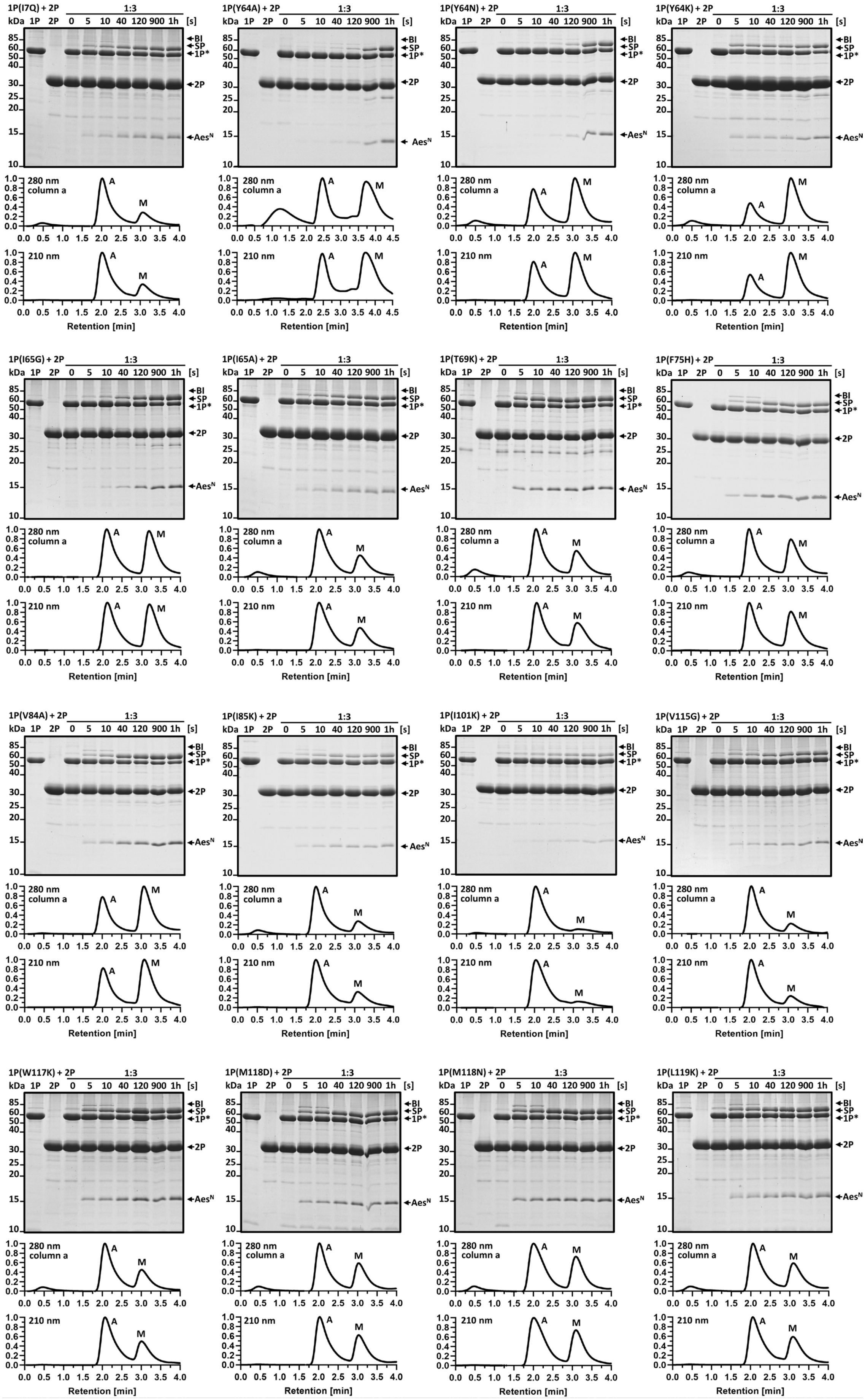

Supplementary Figure 16 Continued on the next page.

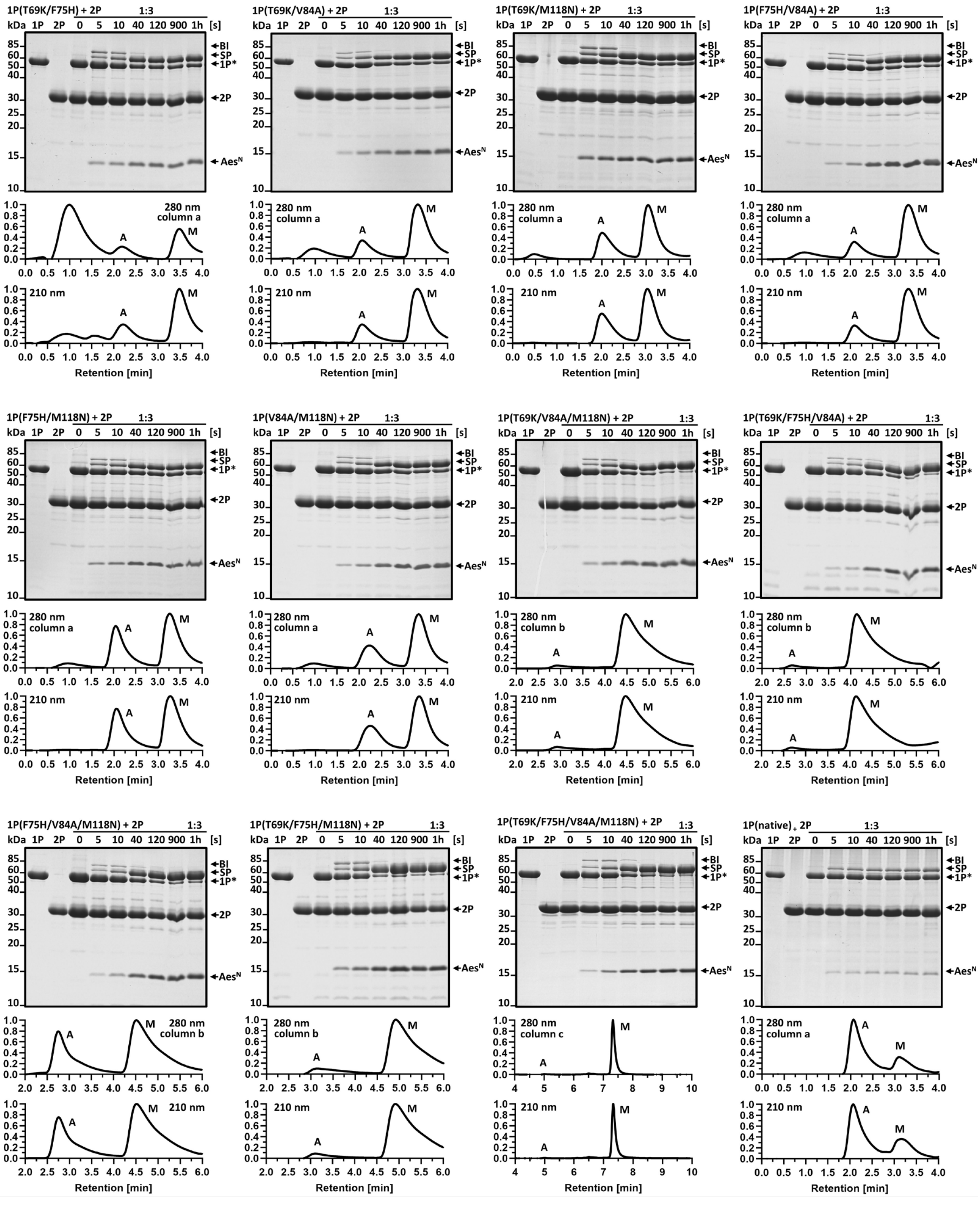

Supplementary Figure 16 Analysis of the aggregation-reducing mutants of MBP-Aes^{N}-H_6 (additional data to Fig. 6a). For each mutant protein *trans*-splicing activity was analyzed using Coomassie-stained SDS-PAGE (upper panel; the experiments were repeated two times) and the monomer/aggregate ratio was determined using analytical SEC and measuring protein absorbance at 280 nm (lower panel). Aggregate (A) and monomeric species (M) are indicated. Note that different SEC columns were used over the course of the project (**columns a**, **b** and **c**). In some cases, an extra signal of unknown origin (marked with asterisk) eluting well ahead of void volume retention time was observed in the 280 nm channel, which, importantly, did not correspond to a protein. For this reason, also the absorbance at 210 nm was monitored and is shown for each sample. PTS reactions were carried out at 37°C and using the indicated mutant of the N-terminal precursor (P_N) with a three-fold molar excess of the C-terminal precursor (P_C) Aes^{C}-GFP (**2P**) (10 µM and 30 µM, respectively). The three SEC columns used were: **column a**, analytical AdvanceBio SEC 120Å 1.9 µm, 2.1 × 150 mm PEEK pre-packed column (Agilent), operated at a flow rate of 0.1 mL/min; **column b**, analytical AdvanceBio SEC 200Å 1.9 µm, 2.1 × 150 mm PEEK pre-packed column (Agilent), operated at a flow rate of 0.1 mL/min; **column c**: analytical AdvanceBio SEC 200Å 1.9 µm, 4.6 × 300 mm pre-packed column (Agilent), operated at a flow rate of 0.35 mL/min. Source data are provided as a Source Data file.

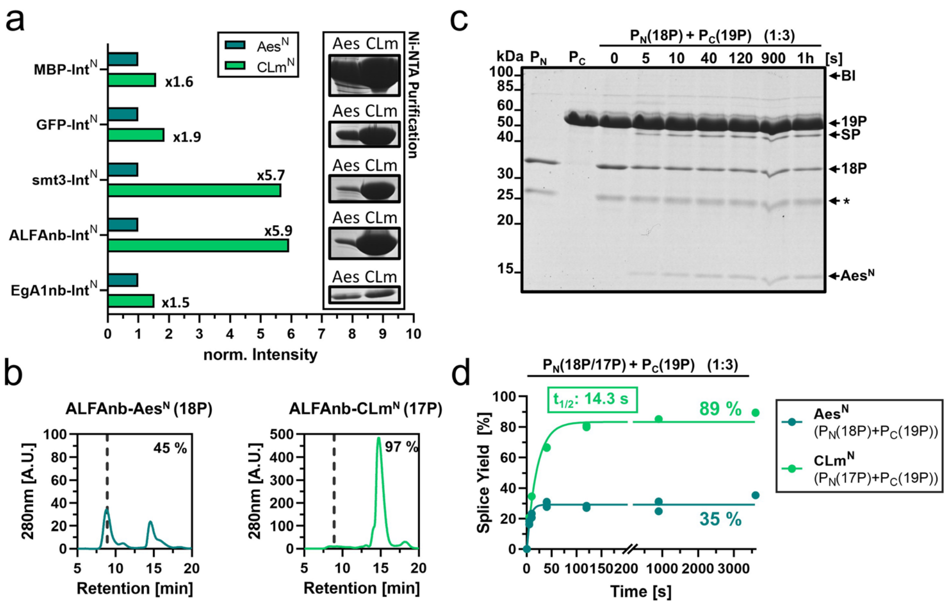

Supplementary Figure 17 The Aes123 PolB1 triple mutant increases the expression yield and protein purity (additional data to Fig. 7a). **a** Densitometric analysis to compare the yields of various purified Aes^N precursor proteins with the corresponding CLm mutants. The intensities were normalized to the Aes^N precursor construct of each pair of proteins. **b** SEC-profiles of the construct ALFAnb-Aes^N-H$_6$ (**18P**) with the native Aes^N sequence compared to the CLm mutant ALFAnb-CLm^N-H$_6$ (**17P**), recorded directly after Ni-NTA affinity purification. Note that the scale of the y-axis was adjusted for a better visualization. **c** SDS-PAGE analysis of the PTS reaction using **18P** together with **19P** used in three-fold molar excess. (5 µM and 15 µM at 37°C). This experiment was repeated two times. **d** Time course of the PTS reactions (also shown in Fig. 7a, b) of either **18P** (blue) or **17P** (green), both used without monomer separation by SEC-purification, together with H$_6$-Smt3-Aes^C-GFP (**19P**) in three-fold molar excess (5 µM and 15 µM, respectively). The reactions were analyzed as pseudo-first order reactions and fitted to a one-phase exponential equation. See Fig. 6b for the SDS-PAGE analysis of the reaction performed with **17P** and **19P** under analogous conditions. Source data are provided as a Source Data file.

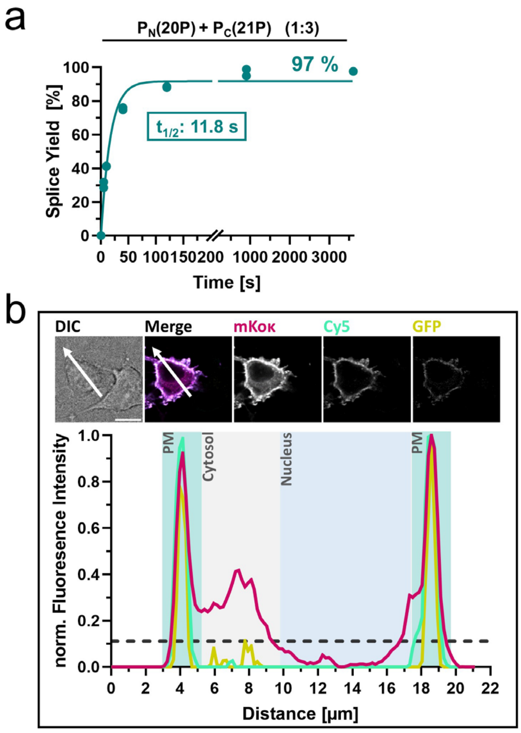

Supplementary Figure 18 Intein-mediated generation of a bispecific nanobody-dimer for cell surface labeling (additional data to Fig. 7c-f). **a** Time course of the PTS reaction (Fig. 7c, d) at 37°C of EgA1nb-CLm^{N}-H$_6$ (**20P**) and H$_6$-CLm^{C}-ALFAnb-SBP (**21P**) (5 µM and 15µM, respectively). **b** Spatial analysis of cellular labeling as described in Fig. 7e, f. Shown are CLSM images of transiently transfected HeLa cells (upper panel) and the quantitative analysis of fluorescence intensity (lower panel) across the cell section along the white arrow. HeLa cells were expressing the extracellular domains of the EGF-receptor (EGFR) exposed on the cell surface together with an intracellular fluorescent marker (mKoκ) (Fig. 7e). Cells were incubated with 250 nM Cy5-labeled **SP(20P-21P)** (cyan) for 10 min at 37°C. Afterwards, 50 nM of GFP-ALFAtag (**22P**) (yellow) was added to the cells and incubated for additional 5 min. The scale bar represents 10 µm. The dashed line marks the maximal background fluorescence in the 488-channel (GFP). Source data are provided as a Source Data file.

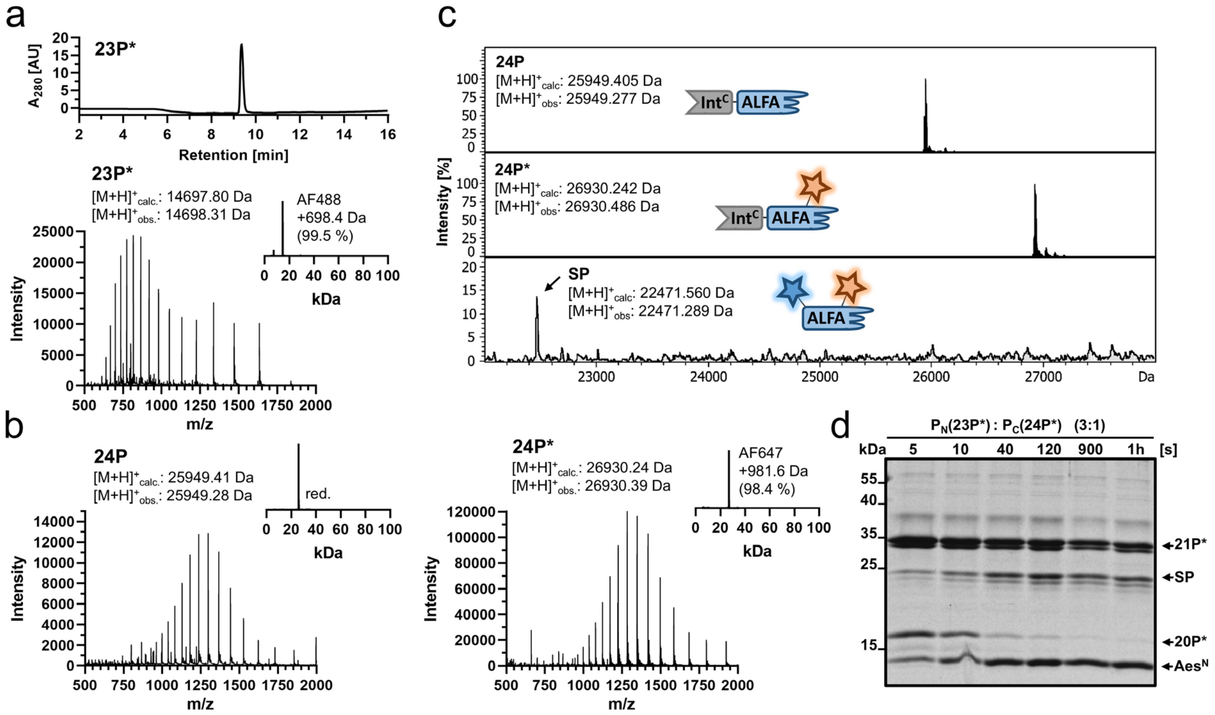

Supplementary Figure 19 Nanobody functionalization using intein-mediated dual thiol-bioconjugation (additional data to Fig. 7g, h). **a** RP-HPLC and ESI-MS analysis of the used AF488-labeled CAD-Aes^{N} (**23P***). **b** ESI-MS analysis of H$_6$-Aes^{C}-ALFAnb(G47C)-SBP (**24P**) before and after labeling by maleimide-AF647 (**24P***). **c** ESI-MS analysis of PTS reaction (shown in Fig. 7g) showing the stepwise dual labeling of the ALFA nanobody of construct H$_6$-Aes^{C}-ALFAnb(G47C)-SBP (**24P**) with the AF488-maleimide by thiol bioconjugation of G47C and subsequent labeling with AF647-labeled tripeptide CAD (N-extein of **23P***) by protein *trans*-splicing. **d** SDS-PAGE analysis via Coomassie staining of the PTS reaction between **23P*** and **24P*** at 37°C (proteins at 5 µM and 15 µM, respectively). Note that the observed labeling efficiency differs between ESI-MS (shown in **c**) and SDS-PAGE analysis (shown in **d**) due to partial thioether cleavage, which removes the maleimide label during sample preparation for SDS-PAGE.[13, 14] This experiment was repeated two times.

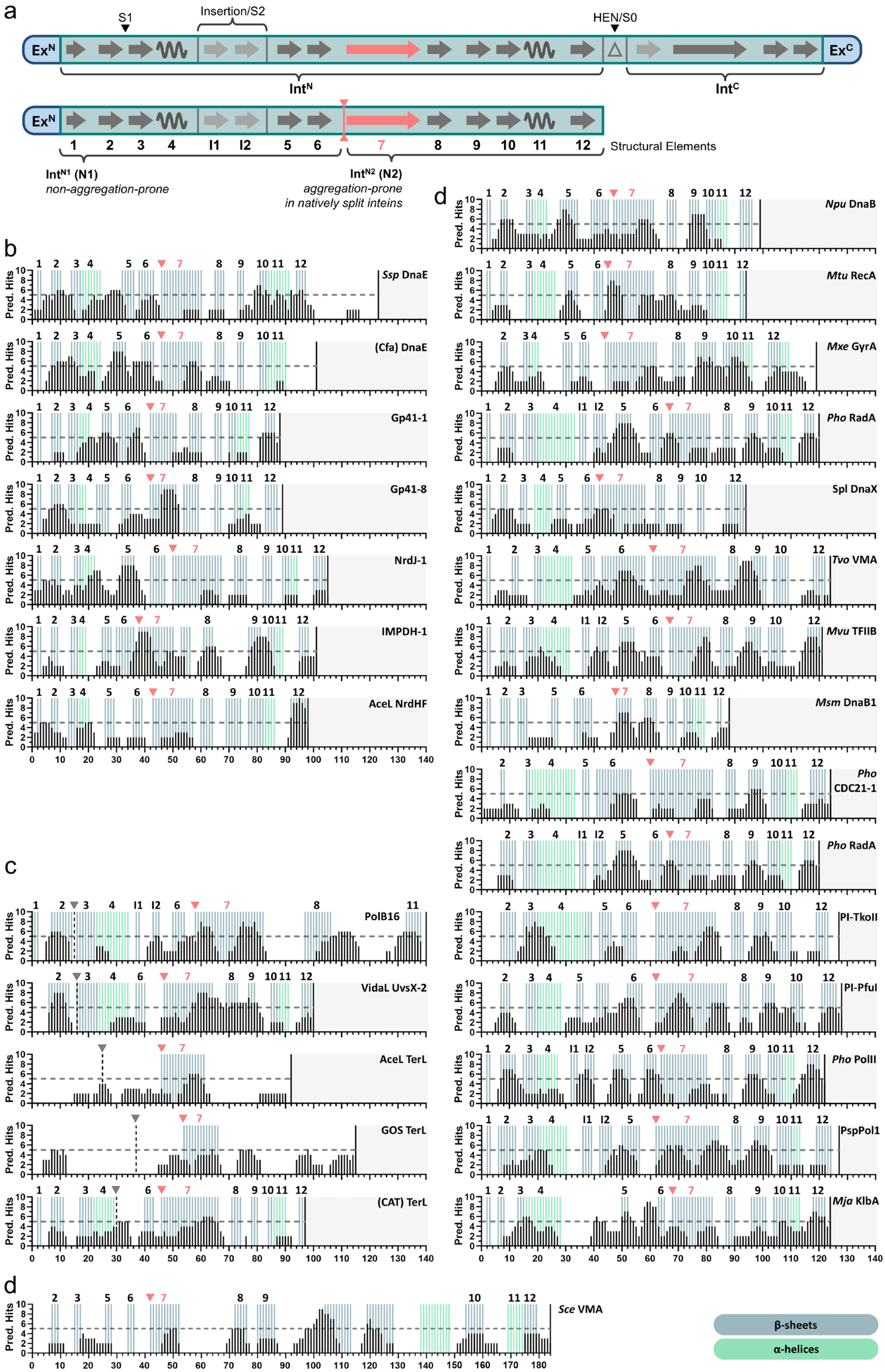
a
S1
Insertion/S2
HEN/S0
Ex^N
Ex^C
Int^N
Int^C
Structural Elements
Int^N1 (N1)
non-aggregation-prone
Int^N2 (N2)
aggregation-prone
in natively split inteins
b
c
d
Pred. Hits
Ssp DnaE
(Cfa) DnaE
Gp41-1
Gp41-8
NrdJ-1
IMPDH-1
AceL NrdHF
PolB16
VidaL UvsX-2
AceL TerL
GOS TerL
(CAT) TerL
Npu DnaB
Mtu RecA
Mxe GyrA
Pho RadA
Spl DnaX
Tvo VMA
Mvu TFIIB
Msm DnaB1
Pho CDC21-1
PI-TkoII
PI-PfuI
Pho PolII
PspPol1
Mja KlbA
Sce VMA
β-sheets
α-helices

Supplementary Figure 20 *In silico* aggregation-prone site prediction of commonly used inteins. **a** Illustration taken from Figure 8a. Shown are conserved secondary structure elements in the minimal intein horseshoe fold for *cis*-inteins (top) and the Int^N fragment of split inteins (bottom). Additionally, one optional insertion of β-strands I1 & I2 is shown that is also present in the Aes split intein. The split positions corresponding to typically split (HEN/S0) and atypically split (S1/S2) inteins are indicated (HEN = intervening homing endonuclease domain).[15, 16] The long β-sheet in the Int^N (structural element 7; marked in red) stretches across an axis of two-fold symmetry in the folded intein structure that has previously been used to define the Int^N N1 and N2 lobes.[6] Here, however, the border between N1 and N2 segments was defined at the beginning of β-sheet 7, indicated by the vertical red line (see main text). **b** Sequence analysis by the consensus prediction algorithm AMYLPRED2[17] of the Int^N part of several commonly used natively split inteins. Note that the structural data of Gp41-8, NrdJ-1 and IMPDH-1 inteins were predicted using AlphaFold3 and Phyre^2 due to the lack of experimental data causing potential artefacts in the *in silico* analysis. **c** Sequence analysis of the canonical Int^N part of commonly used atypically split inteins. Note that the structural data of AceL TerL and GOS TerL inteins were predicted using AlphaFold3 and Phyre^2 due to the lack of experimental data causing potential artefacts in the *in silico* analysis. **d** Sequence analysis of the canonical Int^N part of several commonly used maxi- and mini-inteins. Source data are provided as a Source Data file.

Supplementary Table 5 Inteins and the corresponding canonical Int^{N} sequences used to compare the predicted aggregation tendency.

	Intein	Canonical Int^{N} Sequence	pI
Natively Split Inteins	*Npu* DnaE	CLSYETEILTVEYGLLPIGKIVEKRIECTVYSVDNNGNIYTQPVAQWHDRGEQEVFEYCLEDGSLIRATKDHKFMTVDGQMLPIDEIFERELDLMRVDNLPN	4.39
	Ssp DnaE	CLSFGTEILTVEYGPLPIGKIVSEEINCSVYSVDPEGRVYTQAIAQWHDRGEQEVLEYELEDGSVIRATSDHRFLTTDYQLLAIEEIFARQLDLLTLENIKQTEEALDNHRLPFPLLDAGTIK	4.36
	Cfa DnaE (engineered)	CLSYDTEILTVEYGFLPIGKIVEERIECTVYTVDKNGFVYTQPIAQWHNRGEQEVFEYCLEDGSIIRATKDHKFMTTDGQMLPIDEIFERGLDLKQVDGLP	4.43
	Gp41-1	CLDLKTQVQTPQGMKEISNIQVGDLVLSNTGYNEVLNVFPKSKKKSYKITLEDGKEIICSEEHLFPTQTGEMNISGGLKEGMCLYVKE	5.34
	Gp41-8	CLSLDTMVVTNGKAIEIRDVKVGDWLESECGPVQVTEVLPIIKQPVFEIVLKSGKKIRVSANHKFPTKDGLKTINSGLKVGDFLRSRAK	9.47
	NrdJ-1	CLVGSSEIITRNYGKTTIKEVVEIFDNDKNIQVLAFNTHTDNIEWAPIKAAQLTRPNAELVELEIDTLHGVKTIRCTPDHPVYTKNRGYVRADELTDDDELVVAI	4.80
	IMPDH-1	CFVPGTLVNTENGLKKIEEIKVGDKVFSHTGKLQEVVDTLIFDRDEEIISINGIDCTKNHEFYVIDKENANRVNEDNIHLFARWVHAEELDMKKHLLIELE	4.91
	AceL NrdHF	ALLVGTKVTTKAGDKNIENITLEDEVLQFDMNTKDFSYTNPTKTQKVIRDEIYHFEGAGFDQKVSPNHRMIYEQGGEIKECLAKDFEPSEDKYFIIVE	4.74
	Aes123 PolB1	SVVGDTIIDVSGKKMTIAEFYDSTPDVFMRRNDEARDWVKRVGGKTSLSVNTYSGEVERKNINYIMKHTVKKRMFKIKAGGKEVIVTADHSVMVKRDGKIIDVKPTEMKQTDRVVKWMLT	9.76
Contiguous Inteins (maxi and mini-Inteins)	*Ssp* DnaB	CISGDSLISLASTGKRVSIKDLLDEKDFEIWAINEQTMKLESAKVSRVFCTGKKLVYILKTRLGRTIKATANHRFLTIDGWKRLDELSLKEHIALPR	9.59
	Npu DnaB	CLAGDSLVTLVDSGLQVPIKELVGKSGFAVWALNEATMQLEKAIVSNAFSTGIKPLFTLTTRLGRKIRATGNHKFLTINGWKRLDELTPKEHLALPRNS	9.80
	Mtu RecA	CLAEGTRIFDPVTGTTHRIEDVVDGRKPIHVVAAAKDGTLHARPVVSWFDQGTRDVIGLRIAGGAILWATPDHKVLTEYGWRAAGELRKGDRVA	8.17
	Mxe GyrA	CITGDALVALPEGESVRIADIVPGARPNSDNAIDLKVLDRHGNPVLADRLFHSGEHPVYTVRTVEGLRVTGTANHPLLCLVDVAGVPTLLWKLIDEIKPGDYAVIQRSAFSVDCAGFAR	5.43
	Mvu TFIIB	SVDYSEPIIIKEKGEIKVVKIGELIDEIIKNSKNVRKDGILEIARCKDVEVIAFDSNYKFKFMPVSEVSRHPVSEMFEIVVEGNKKVRVTGSHSVFTVKDNEVVPIRVDDLRVGDILVLAK	6.14
	Msm DnaB1	ALALDTPLPTPSGWTTMGDVAVGDHLLGPDGEPTRVVADTDVMLGRPCYVVEFSDGTAIVADAQHQWPTEHGVRITANLRAGMHTVVS	4.59
	Pho RadA	CFARDTEVYYENDTVPHMESIEEMYSKYASMNGELPFDNGYAVPLDNVFVYTLDIASGEIKKTRASYIYREKVEKLIEIKLSSGYSLKVTPSHPVLLFRDGLQWVPAAEVKPGDVVVGVR	4.99
	Pho CDC21-1	AVDYDTEVLLGDGRKRKIGEIVEEAIKKAEKEGKLGRVDDGFYAPINLELYALDVRTLKVRKVKADIAWKRTTPEKMLRIRTKRGREIRVTPTHPFFTLEEGRIKTKKAYELKVGEKIATPREE	9.69
	Pho PolII	CFPGDTRILVQINGTPQRVTLKELYELFDEEHYESMVYVRKKPKVDIKVYSFNPEEGKVVLTDIEEVIKAPATDHLIRFELELGSSFETTVDHPVLVYENGKFVEKRAFEVREGNIIIIIDE	4.82
	Spl Dnax	ALTGDALILSDRGWLRIDDPTLQECRVLSYNESTQQWEWQQVLRWLDQGVRETWKIKTFQTEIKCTGNHLIRTDKGWIKAANITPKMKILSPEI	8.03
	Mja KlbA	ALAYDEPIYLSDGNIINIGEFVDKFFKKYKNSIKKEDNGFGWIDIGNENIYIKSFNKLSLIIEDKRILRVWRKKYSGKLIKITTKNRREITLTHDHPVYISKTGEVLEINAEMVKVGDYIYIPK	9.28
	PI-PfuI	CIDGKAKIIFENEGEEHLTTMEEMYERYKHLGEFYDEEYNRWGIDVSNVPIYVKSFDPESKRVVKGKVNVIWKYELGKDVTKYEIITNKGTKILTSPWHPFFVLTPDFKIVEKRADELKEGDILIGGM	5.28
	PI-TkoII	SILPEEWLPVLEEGEVHFVRIGELIDRMMEENAGKVKREGETEVLEVSGLEVPSFNRRTNKAELKRVKALIRHDYSGKVYTIRLKSGRRIKITSGHSLFSVRNGELVEVTGDELKPGDLVAVPRRLE	6.83
	PspPol-1	SILPEEWVPLIKNGKVKIFRIGDFVDGLMKANQGKVKKTGDTEVLEVAGIHAFSFDRKSKKARVMAVKAVIRHRYSGNVYRIVLNSGRKITITEGHSLFVYRNGDLVEATGEDVKIGDLLAVPRSVN	9.88
	Sce VMA	CFAKGTNVLMADGSIECIENIEVGNKVMGKDGRPREVIKLPRGRETMYSVVQKSQHRAHKSDSSREVPELLKFTCNATHELVVRTPRSVRRLSRTIKGVEYFEVITFEMGQKKAPDGRIVELVKEVSKSYPISEGPERANELVESYRKASNKAYFEWTIEARDLSLLGSHVRKATYQTYAPILY	9.32
	Tvo VMA	CVSGETPVYLADGKTIKIKDLYSSERKKEDNIVEAGSGEEIIHLKDPIQIYSYVDGTIVRSRSRLLYKGKSSYLVRIETIGGRSVSVTPVHKLFVLTEKGIEEVMASNLKVGDMIAAVAESASE	6.17
Atypically Split Inteins	VidaL UvsX-2	CLPKEAVVQIRLTKKGMIEEKKVTVQELRELYLSGEYTIEIDTPDGYQTIGKWFDKGVLSMVRVATATYETVCAFNHMIQLADNTWVQACELDVGVDIQT	4.75
	PolB-16	SVHGKTHVFIRSIKNMQEAKIDIKSLYDSLAKKYDVQHKNSYEVIYPKGYEIKVLGNKYVKLVAMSRHKTQKHLVKIVVKSEKTIDSLDPIRQKSLLKKQDEVVVTTDHICMVYNDDHFFENVNAKNLKVGNYVSVYDEA	9.33
	AceL TerL	CVYGDTMVETEDGKIKIEDLYKRLAMFRTNTNNIKILSPNGFSNFNGIQKVERNLYQHIIFDDDTEIKTSINHPFGKDKILARDVKVGDYLN	6.11
	GOS TerL	SISQESYINIEVNGKVETIKIGDLYKKLSFNERKFNEMKLPESVVKNNINLKIETPYGFENFYGVNKIKKDKYIHLEFTNGEKLKCSLDHPLSTIDGIVKAKDLDKYTEVYTKFG	8.51
	CAT TerL (engineered)	CLSGDTMIEILDDDGIIQKISMEDLYQRLAMFKLNTKNIKVLTPSGFKSFSGIQKVYKPFYHHIIFDDGSEIKCSDNHSFGKDKIKASTIKVGDYLQ	7.00

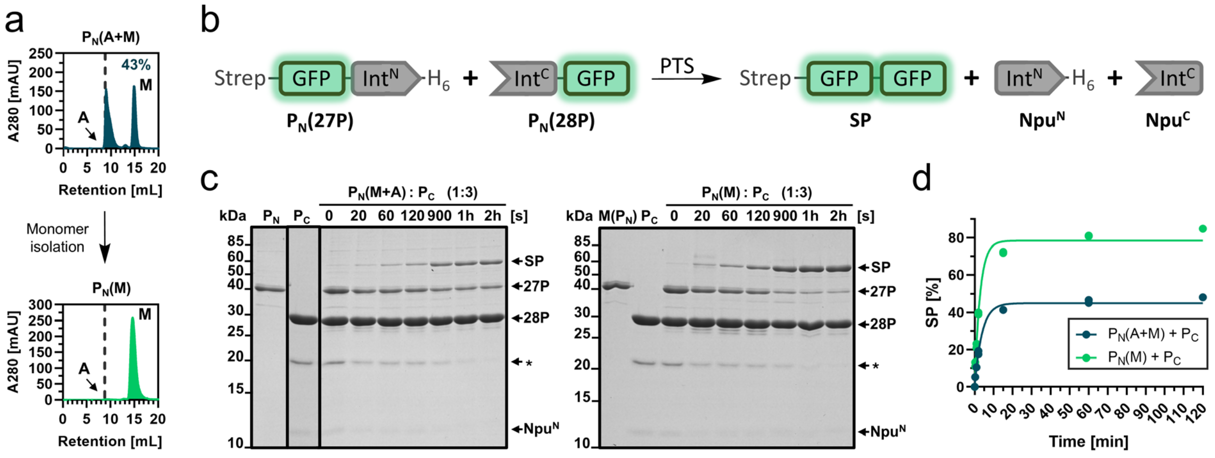

Supplementary Figure 21 Int^N aggregation into inactive species in the natively split *Npu* DnaE intein. **a** SEC-analysis of **27P** before (upper panel) and after (lower panel) monomer isolation. These two preparations were used for the reactions in **c**. **b** Scheme of the PTS reaction using Strep-GFP-Npu^N (**27P**) and Npu^C-GFP (**28P**). **c** Coomassie-stained SDS-PAGE analysis of the PTS reaction (under reducing conditions) with **27P** and **28P** used in three-fold molar excess (3 µM and 9 µM, 37°C) without prior SEC-purification (left panel) and after monomer isolation of **27P** (P_N(M(**27P**)) (right panel). The experiments were repeated two times. **d** Time-course of the PTS reactions in **c** fitted to a one-phase exponential equation. P_N(**27P**) = 40.8 kDa, P_C(**28P**) = 31.4 kDa, Npu^N = 12.8 kDa, Npu^C = 4.1 kDa, SP = 56.3 kDa. SP = splice product. Source data are provided as a Source Data file.

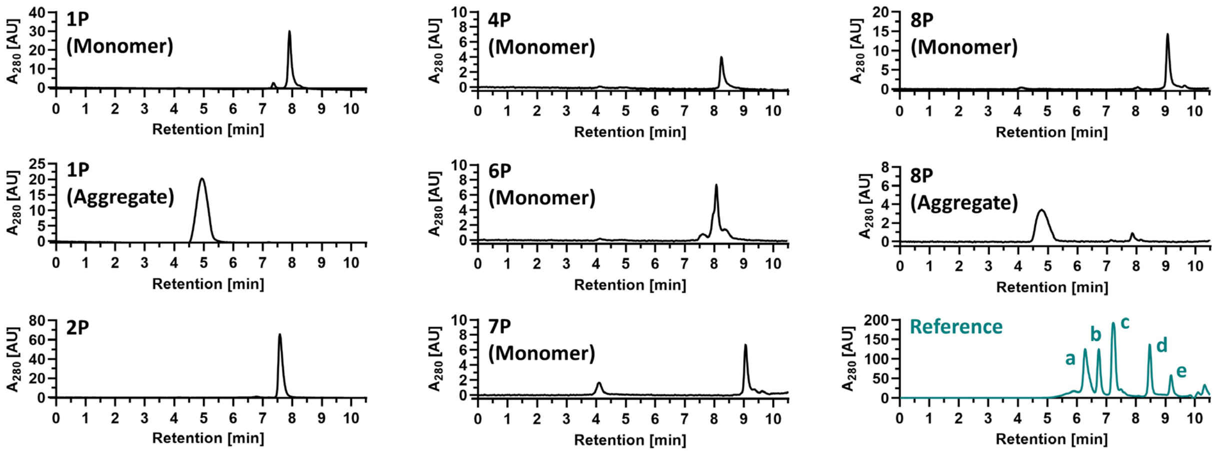

Supplementary Figure 22 SEC analysis of the purified and isolated monomeric or aggregated proteins used throughout this study. The reference SEC UV-chromatogram indicates the usual retention time of globular proteins between 12 kDa and 200 kDa while the aggregated species show a retention time of 4 – 5.5 min. Proteins were analyzed on AdvanceBio SEC 200Å 1.9 µm, 4.6 × 300 mm pre-packed column (Agilent) at a follow rate of 0.35 mL/min. (a: β-amylase (200 kDa), b: alcohol dehydrogenase (150 kDa), c: bovine serum albumin (66 kDa), d: carbonic anhydrase (29 kDa), e: cytochrome c (12.4 kDa). Source data are provided as a Source Data file.

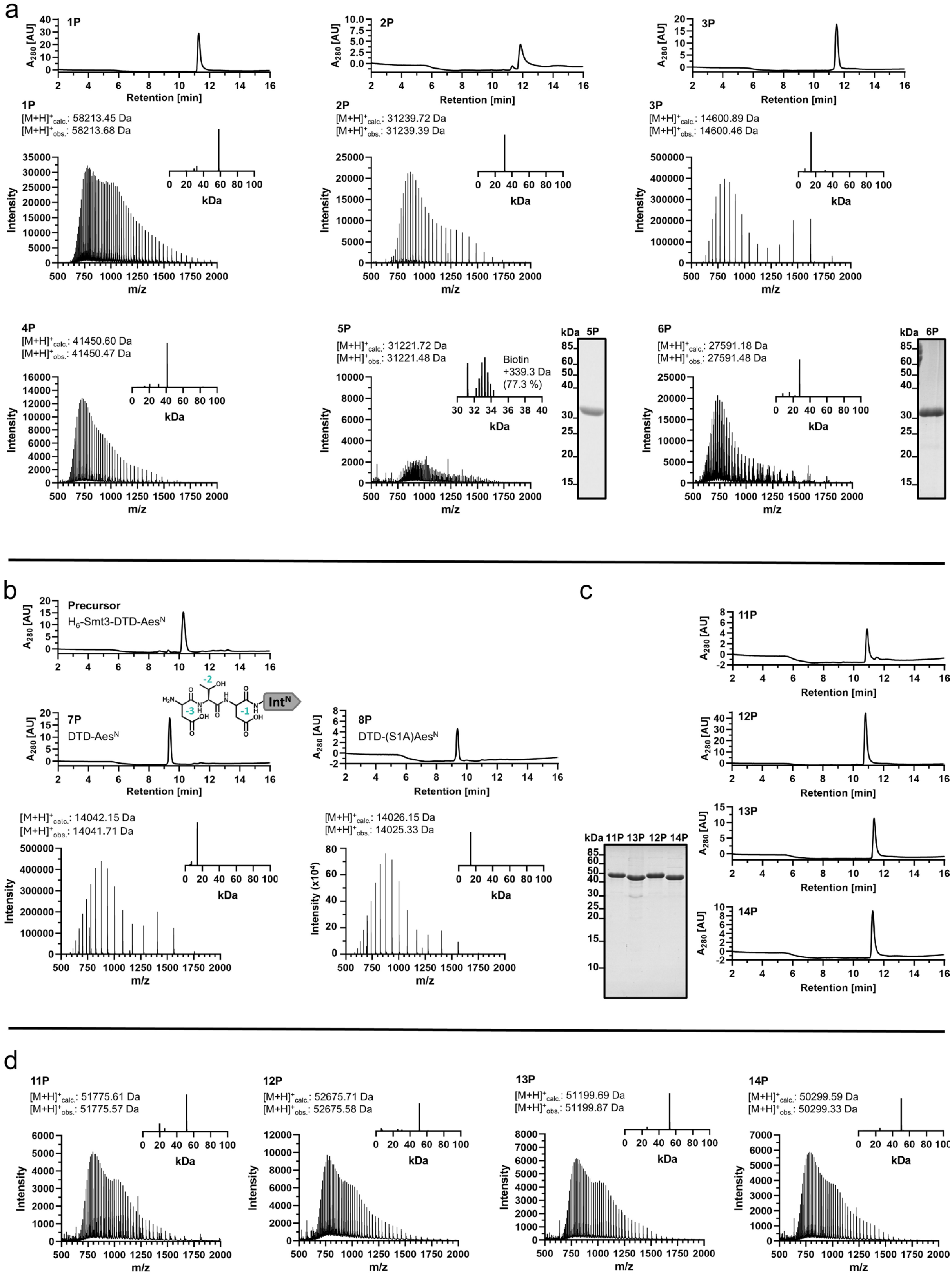

Supplementary Figure 23 Continued on next page.

e

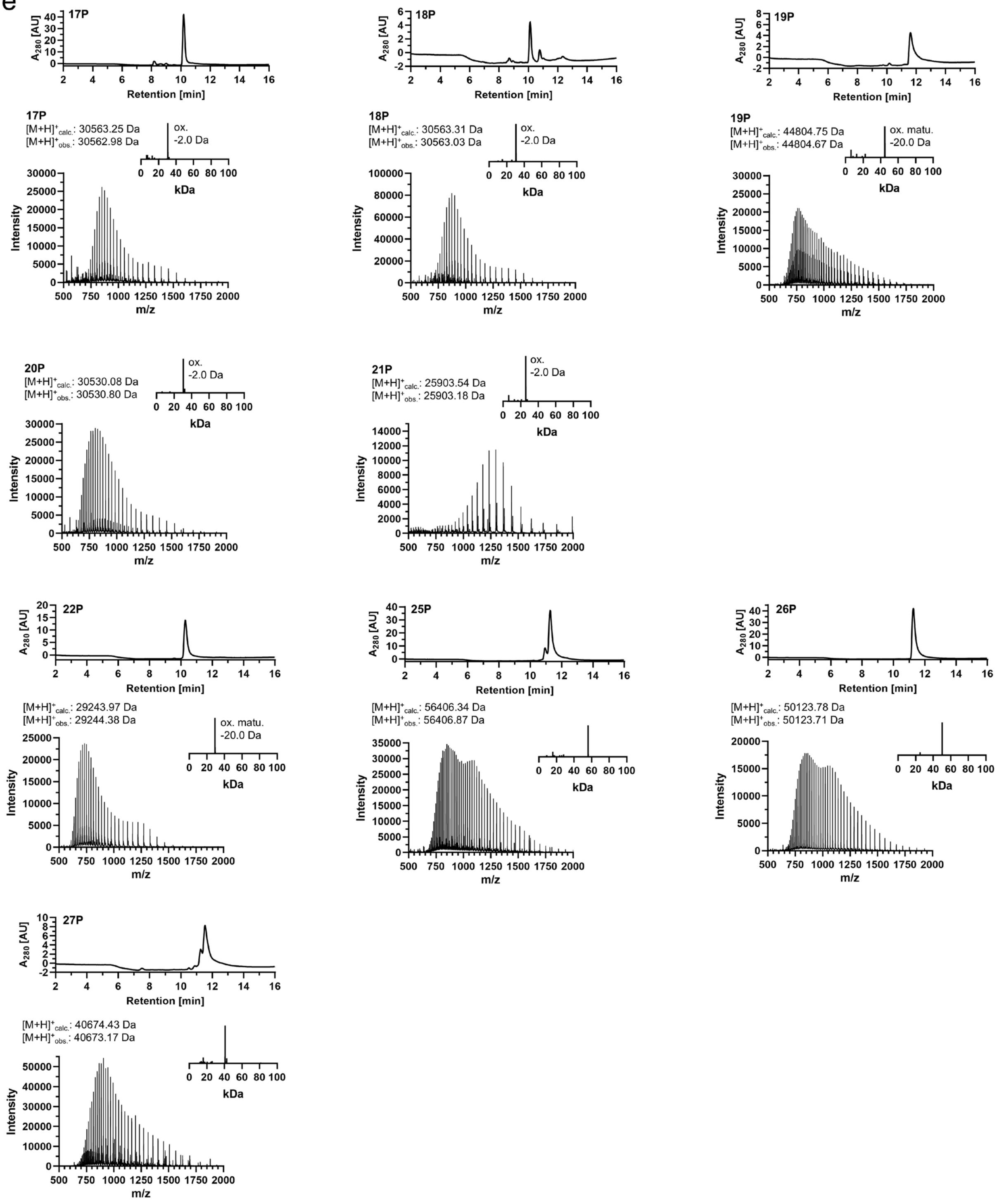

Supplementary Figure 23 Analytics to the used constructs. **a** RP-HPLC UV-chromatograms (280 nm) to monitor purity of purified constructs **1P**, **2P** and **3P**. ESI-MS analysis of purified constructs **1P**, **2P**, **3P**, **4P**, **5P** (labeled via NHS-C2-Biotin) and **6P**. Shown are the original MS and the deconvoluted spectra (insets). SDS-PAGE analysis to monitor purity of purified constructs **5P** (labeled via NHS-C2-Biotin) and **6P**. **b** RP-HPLC UV-chromatograms to monitor purity of purified constructs **7P**, its precursor construct with a H_6-Smt3 tag (exemplary shown) and **8P**. ESI-MS analysis of purified constructs **7P** and **8P**. **c** RP-HPLC UV-chromatograms and SDS-PAGE analysis to monitor purity of purified constructs **11P**, **12P**, **13P** and **14P**. **d** ESI-MS analysis of purified constructs **11P**, **12P**, **13P** and **14P**. (E) RP-HPLC UV-chromatograms to monitor purity of purified constructs **17P**, **18P**, **19P**, **22P**, **25P**, **26P** and **27P**. ESI-MS analysis of the used constructs to prove protein identity and redox state of the purified constructs **17P**, **18P**, **19P**, **20P**, **21P**, **SP(20P-21P)-Cy5**, **22P**, **25P**, **26P** and **27P** (ox.: oxidized; matu.: chromophore maturation within GFP).

Supplementary References

1. Bhagawati, M. et al. A mesophilic cysteine-less split intein for protein trans-splicing applications under oxidizing conditions. *Proc Natl Acad Sci U S A* **116**, 22164-22172 (2019).
2. Jedlitzke, B. & Mootz, H.D. A Light-Activatable Photocaged Variant of the Ultra-High Affinity ALFA-Tag Nanobody. *Chembiochem* **23**, e202200079 (2022).
3. Brenzel, S., Kurpiers, T. & Mootz, H.D. Engineering artificially split inteins for applications in protein chemistry: biochemical characterization of the split Ssp DnaB intein and comparison to the split Sce VMA intein. *Biochemistry* **45**, 1571-1578 (2006).
4. Zettler, J., Schütz, V. & Mootz, H.D. The naturally split *Npu* DnaE intein exhibits an extraordinarily high rate in the protein *trans*-splicing reaction. *FEBS Lett* **583**, 909-914 (2009).
5. Mukherjee, S., Shukla, A. & Guptasarma, P. Single-step purification of a protein-folding catalyst, the SlyD peptidyl prolyl isomerase (PPI), from cytoplasmic extracts of Escherichia coli. *Biotechnol Appl Biochem* **37**, 183-186 (2003).
6. Shah, N.H., Eryilmaz, E., Cowburn, D. & Muir, T.W. Naturally split inteins assemble through a "capture and collapse" mechanism. *J Am Chem Soc* **135**, 18673-18681 (2013).
7. Tiwari, P.B., Wang, X., He, J. & Darici, Y. Analyzing surface plasmon resonance data: choosing a correct biphasic model for interpretation. *Rev Sci Instrum* **86**, 035001 (2015).
8. Dunker, A.K. et al. Intrinsically disordered protein. *J Mol Graph Model* **19**, 26-59 (2001).
9. Williams, C.J. et al. MolProbity: More and better reference data for improved all-atom structure validation. *Protein Sci* **27**, 293-315 (2018).
10. Beyer, H.M., Mikula, K.M., Kudling, T.V. & Iwai, H. Crystal structures of CDC21-1 inteins from hyperthermophilic archaea reveal the selection mechanism for the highly conserved homing endonuclease insertion site. *Extremophiles* **23**, 669-679 (2019).
11. Pasch, T. et al. Structural and biochemical analysis of a novel atypically split intein reveals a conserved histidine specific to cysteine-less inteins. *Chem Sci* **14**, 5204-5213 (2023).
12. Dodson, G. & Wlodawer, A. Catalytic triads and their relatives. *Trends Biochem Sci* **23**, 347-352 (1998).
13. Zhang, C. et al. Loss of PEG chain in routine SDS-PAGE analysis of PEG-maleimide modified protein. *Electrophoresis* **36**, 371-374 (2015).
14. Cooper, C.E. et al. Stability of Maleimide-PEG and Mono-Sulfone-PEG Conjugation to a Novel Engineered Cysteine in the Human Hemoglobin Alpha Subunit. *Front Chem* **9**, 707797 (2021).
15. Sun, W., Yang, J. & Liu, X.Q. Synthetic two-piece and three-piece split inteins for protein trans-splicing. *J Biol Chem* **279**, 35281-35286 (2004).
16. Thiel, I.V., Volkmann, G., Pietrokovski, S. & Mootz, H.D. An atypical naturally split intein engineered for highly efficient protein labeling. *Angew Chem Int Ed Engl* **53**, 1306-1310 (2014).
17. Tsolis, A.C., Papandreou, N.C., Iconomidou, V.A. & Hamodrakas, S.J. A consensus method for the prediction of 'aggregation-prone' peptides in globular proteins. *PLoS One* **8**, e54175 (2013).
