# Supplementary material for: A cysteine-less and ultra-fast split intein rationally engineered from being aggregation-prone to highly efficient in protein trans-splicing
Source: Nat Commun. 2025 Mar 19;16:2723. doi: 10.1038/s41467-025-57596-x (PMC11923092; doi:10.1038/s41467-025-57596-x)
Supplement: Supplementary file 2 — Description of Additional Supplementary Information [file 41467_2025_57596_MOESM2_ESM.docx]

**Description of Additional Supplementary Files**

File Name: Supplementary Data 1

Description: List of sequences of recombinantly produced proteins. The marked sequences either indicate the Nterminal proteolytically cleavable H6- Smt3 tag or the C-terminal chemically cleavable GyrA-CBD tag.

File Name: Supplementary Data 2

Description: List of oligonucleotides used for cloning to introduce the aggregation-reducing single mutations.

File Name: Supplementary Data 3

Description: Software output data for LC-MS2 analysis of carbene labelled tryptic peptides.
